# Characterizing locomotor behavior variability in commercial broiler flocks using large-scale video tracking

**DOI:** 10.1016/j.psj.2026.107363

**Published:** 2026-06-30

**Authors:** Noslen Hernández, Sylvain L’Hermite, Pauline Créach, Didier Concordet

**Affiliations:** a Université de Toulouse, ENVT, INRAE, INTHERES, Toulouse, France; b ITAVI, 7 rue du Faubourg Poissonnière, 75009, Paris, France

**Keywords:** Broiler, Deviation-based monitoring, Commercial farm, Video tracking, Movement analysis

## Abstract

The surveillance of broiler chicken welfare requires objective and scalable approaches capable of capturing behavioral dynamics under commercial production conditions. This study characterized locomotor behavior in broiler flocks reared under commercial conditions using large-scale video observations. A multi-camera system was deployed across four commercial farms, encompassing 12 flocks and more than 14,500 h of recordings. Broilers were automatically detected and tracked to extract spatiotemporal movement descriptors. Three complementary indicators were analyzed: the trajectory segment-based proportion of inactivity, the mean speed of active trajectory segments, and collective movement events reflecting transient synchronization of motion. Statistical models were used to estimate expected temporal trajectories and prediction intervals for each indicator across age, daily activity period, sex, and farm. The results revealed structured patterns of locomotor organization across production cycles and farms, allowing the expected range of behavioral variability under routine commercial conditions to be quantified. When applied to observations recorded under non-routine conditions, deviations from these reference ranges occurred more frequently, indicating departures from typical flock locomotor dynamics. These findings demonstrate that large-scale video observations can provide quantitative reference patterns for broiler locomotor behavior under commercial conditions. Establishing such baselines offers a practical foundation for behavioral monitoring approaches aimed at identifying atypical flock activity patterns in commercial poultry production systems.

## Introduction

The assessment of broiler chicken welfare remains a central challenge in modern poultry production, driven by ethical expectations and the need for sustainable and efficient management practices. Under commercial conditions, welfare is shaped by complex interactions between animals, their environment, and management routines, making continuous and objective evaluation difficult at flock scale. Automated monitoring technologies are therefore increasingly viewed as essential tools for achieving non-invasive, high-resolution behavioral assessment in commercial systems (Ben Sassi et al., 2016; Okinda et al., 2020).

Among these technologies, video tracking and computer vision systems have enabled precise quantification of locomotor behavior at both individual and flock levels (Aydin et al., 2010; Dawkins et al., 2009, 2012). Such systems reduce the need for direct human observation, minimize disturbance, and provide continuous behavioral data over extended periods (Aydin and Berckmans, 2016; Neethirajan, 2022). Recent advances in artificial intelligence have further expanded these capabilities. Deep learning methods now allow reliable detection, tracking, and behavioral characterization of broilers even under variable lighting and stocking density conditions (Chen et al., 2023; Campbell et al., 2024; Jaihuni et al., 2023; Nasiri et al., 2024; Siriani et al., 2022; Yang et al., 2024). Together with the rapid evolution of object detection architectures such as the YOLO (You Only Look Once) family (Tian et al., 2025; Wang et al., 2024, 2025), these developments have made scalable monitoring of large commercial flocks technically feasible.

Most existing automated approaches have focused on identifying specific welfare problems, such as lameness, footpad dermatitis, or thermal stress, by associating predefined behavioral or physiological cues with known impairments (Aydin, 2017; Dawkins et al., 2017; Sgavioli et al., 2023). While such targeted detection strategies are valuable, they rely on prior knowledge of causal links between behavior and welfare outcomes, which are often difficult to establish robustly under commercial production conditions and may remain incomplete, context-dependent, or farm-specific. Moreover, no consensus currently exists regarding a specific or universal behavioral signature of impaired welfare in broiler chickens, particularly under commercial production conditions.

An alternative strategy consists of first characterizing the range of behavioral variability expressed under routine commercial conditions, and then identifying structured deviations from this reference as potential signals of altered system functioning. Rather than diagnosing predefined impairments, this approach aims to describe expected locomotor organization across time, farms, and production flocks, thereby providing quantitative reference ranges against which new observations can be evaluated. This deviation-based logic aligns with recent perspectives emphasizing behavioral adaptability and context-sensitive monitoring as central components of welfare surveillance (Dawkins, 2024).

The present study was conducted under fully commercial rearing conditions, involving broilers monitored across multiple farms, production flocks, and camera setups. This design captures the biological and managerial variability inherent to real-world production systems and results in hierarchically structured data at the farm, flock, and camera levels. Although such heterogeneity introduces modelling challenges, it ensures that the proposed framework reflects realistic operational conditions. Previous large-scale monitoring efforts have similarly emphasized the importance of integrating cross-site variability to enhance robustness and practical relevance (Campbell et al., 2024; Dawkins, 2024; Doornweerd et al., 2024).

In this study, neural-network-based video tracking was used to monitor individual broilers and extract locomotor descriptors. Three complementary behavioral indicators were analyzed: the trajectory segment-based proportion of inactivity, the mean speed of active trajectory segments, and the occurrence of collective movement episodes reflecting transient synchronization of motion. Together, these metrics capture interrelated aspects of inactivity, movement intensity, and group-level coordination, providing a multidimensional representation of flock locomotor dynamics under commercial conditions. Unlike optical-flow approaches, which quantify global pixel-level displacement within the image, this framework derives movement descriptors from tracked individual trajectories before aggregation. This allows routine-condition variability to be characterized through distinct, interpretable locomotor components rather than a single global motion signal, and deviations to be evaluated relative to covariate-adjusted reference ranges.

The modelling framework relies on generalized linear models fitted to time-structured behavioral data, enabling estimation of expected temporal trajectories and the definition of prediction intervals representing normal variability under routine conditions. By identifying deviations from these model-derived reference ranges, the system generates behavioral alerts without requiring prior labelling of welfare states or assumptions about specific causal mechanisms.

This combination of individual- and group-level metrics constitutes a data-driven strategy for automated behavioral surveillance in commercial broiler systems. By shifting the focus from classifying specific welfare impairments to quantifying routine-condition variability and detecting departures from expected locomotor organization, the proposed approach establishes interpretable reference patterns while explicitly acknowledging the limits of causal inference in large-scale welfare monitoring.

## Materials and methods

### Farm description and video system

The study was conducted on four commercial broiler farms situated in Brittany, France, each housing Ross 308 broiler chickens that arrived on the farms as one-day-old chicks. On each farm, three consecutive flocks were monitored between February and September 2024, resulting in a total of 12 monitored flocks. The farms operated under commercial conditions corresponding to a sex-segregated heavy broiler production system, with environmentally controlled buildings and separate male and female rearing zones.

**Fig. 1:**
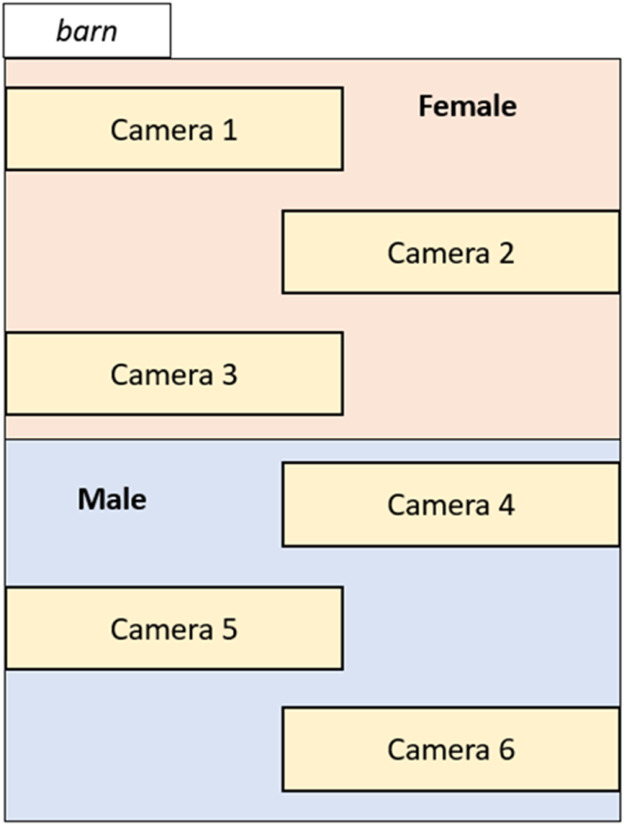
Schematic representation of a typical farm building layout in this study, showing the division between male and female zones and the positioning of the six overhead cameras used for video recording. Each camera covers a fixed sampling area of approximately 8.35 m by 3.33 m, including multiple feed and water lines. These fixed camera views were used to sample representative areas within each sex-specific zone, rather than to monitor the entire house surface.

Each building was equipped with six 4 K resolution cameras (DS-2CD2086G2-IU, 2.8 mm; 3840 × 2160 pixels) mounted at a height of 3.5 m and oriented perpendicularly to the ground, three cameras covering the male area and three covering the female area. Each camera covered an area of approximately 8.35 × 3.33 m, including two to three feed lines, three to five water lines, and adjacent wall sections. Depending on the bird age, between 500 and 1000 broilers were visible per camera. The camera system therefore sampled fixed zones within each rearing area rather than covering the entire house surface. Fig. 1 illustrates the configuration of camera placement and sex-segregated areas within a typical building. Housing surfaces ranged from 960 to 2000 m^2^, with total flock sizes between approximately 18,697 and 39,780 birds. Stocking density varied from 17.34 to 21.84 broilers/m^2^, and the maximum live-weight stocking density reached 42.63 kg/m² and was observed in the female area before female thinning.

Video recordings were scheduled for three daily 90-minute periods defined relative to each farm’s light schedule: post-lights-on, mid-light period, and pre-lights-off. These periods were used to capture the main daily fluctuation in flock activity while standardizing recordings with respect to the light cycle rather than fixed clock times, and to maintain a feasible volume of video data for storage, transfer, and AI-based processing. Data collection started at chick placement and continued until day 45, resulting in a total of 14,580 h of footage covering variations across farms, flocks, sex, and time of day.

### Video processing pipeline

All video footage first underwent geometric correction to remove distortions introduced by the cameras’ wide-angle lenses. Radial and tangential distortions were corrected using the Brown–Conrady model, as implemented in OpenCV 4.10 (Conrady, 1919; Zhang, 2000). For each camera, intrinsic parameters and distortion coefficients were estimated through a standard calibration procedure: a checkerboard pattern was moved throughout the entire field of view and imaged from multiple angles and positions. These calibration images allowed quantifying how straight checkerboard lines were warped by the optics, enabling accurate correction of lens deformation across the whole monitored area.

A dataset of 86,924 annotated broiler instances from 841 annotated images or image patches was used to train the detection model. This dataset was assembled for a computer-vision task and selected to cover visual variability relevant for broiler detection, including variation in bird size, posture, density, lighting, background, and image quality. It was used only for detector training, and was not intended to constitute a balanced sample of all monitored flocks. The YOLOv8 architecture was selected based on preliminary comparisons of accuracy and processing cost. Training was performed using the Ultralytics implementation (version 8.3.80) in Python 3.11.5, starting from a pretrained lightweight model and fine-tuning it for a single class (“broiler”). The training ran for up to 500 epochs with an early-stopping patience of 100 epochs, a batch size of 1, and an image size of 1920 px. Default Ultralytics hyperparameters were used, including momentum = 0.937, weight decay = 0.0005, warm-up for 3 epochs, and standard data augmentation (hue–saturation–value shifts, scaling, translation, and horizontal flips). Training was executed on an NVIDIA RTX A4500 GPU. The model was evaluated on a validation set of 10 images containing 5,766 annotated birds.

After detection, birds were tracked across frames using BoT-SORT (Aharon et al., 2022), which combines appearance embeddings with Kalman filtering and matching heuristics to maintain short-term trajectory continuity. Videos were processed at 15 frames per second, providing sufficient temporal resolution for estimating short-term movement dynamics. The tracker output consisted of trajectory segments, each with an identifier valid over the period during which the bird was continuously tracked, together with frame index, image-plane coordinates (x,y), and bounding-box dimensions.

**Fig. 2:**
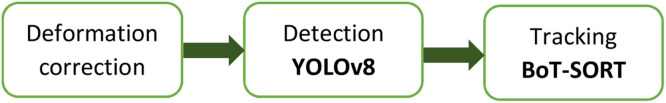
Overview of the video processing pipeline used in this study, including geometric distortion correction based on per-camera geometric calibration, broiler detection using a YOLOv8 model, and individual tracking with the BoT-SORT algorithm. The resulting dataset provides temporally resolved spatial positions for each identified bird, enabling the computation of locomotion metrics.

Long-term identity preservation or re-identification after a bird left and re-entered the camera field of view was not required for the locomotion indicators used in this study. These indicators relied on short-term displacement dynamics aggregated within 90-min observation units. The duration of retained trajectory segments and the number of retained segments contributing to each observation unit are summarized in Supplementary Tables S2–S3 and Supplementary Figures S1–S2. The resulting structured tracking dataset therefore enabled the reconstruction of trajectory segments and the computation of the locomotor indicators used in the subsequent analyses. A schematic overview of the full processing pipeline is shown in Fig. 2.

### Event logging and construction of the routine-condition training set

**Fig. 3:**
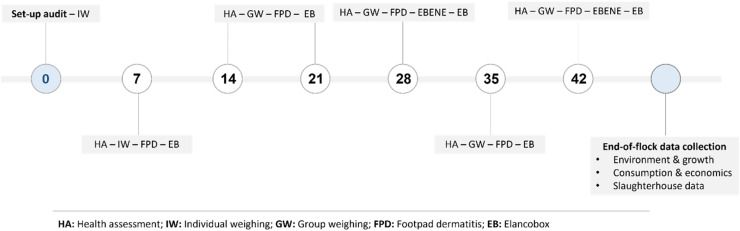
Health and welfare monitoring protocol implemented during the production cycle. The time line summarizes the schedule of veterinary visits, health assessments, and data collection activities conducted on each farm throughout the rearing period. At predefined days of life (0, 7, 14, 21, 28, 35 and 42), standardized evaluations were performed, including health assessment (HA), fecal examination using Elancobox (EB), individual or group weighing (IW, GW), footpad dermatitis scoring (FPD), and welfare assessment using the EBENE method. End-of-flock data collection included environmental, growth, consumption, slaughterhouse, and economic data. This monitoring framework ensured consistent contextual monitoring and provided background information for the interpretation of behavioral measurements.

A routine health and production monitoring program was implemented on each farm, including regular visits by farm staff and veterinarians (Fig. 3). The primary purpose of this monitoring was to provide contextual information on conditions that may transiently alter locomotor expression or compromise the interpretation of video-derived movement descriptors.

Reported incidents were used to define exclusion windows and to construct a routine-condition training dataset for baseline construction. Exclusion criteria targeted periods incompatible with locomotor baseline construction, including marked environmental disruptions, technical irregularities, management-related disturbances, or suspected health events recorded during routine farm and veterinary monitoring (Table 1). Observations falling within these exclusion windows were removed from the routine-condition training dataset and assigned to the non-routine dataset. Importantly, exclusion windows were defined from farm and veterinary monitoring records and were not based on the apparent locomotor behavior of the flock, video-derived locomotor indicators, model residuals, or alert status. The event categories underlying this assignment are summarized in Supplementary Table S1.

**Table 1:** Examples of contextual event records used to assign observations to the routine-condition or non-routine datasets. The table provides examples of records available during routine farm monitoring and indicates whether observations associated with these records were assigned to the non-routine dataset. It is not intended as an exhaustive list of all event categories. A detailed summary of the event categories included in the non-routine dataset is provided in Supplementary Table S1.

**Examples of contextual event records**	**Observations assigned to non-routine dataset**
Abnormal indoor climate (too hot, high CO₂, etc.)	Yes
GRIT addition	Yes
Technical issue (e.g., insufficient water flow)	Yes
Suspected health or mortality event	Yes
Disturbance (e.g., farmer entry, construction work)	Yes
Poor litter quality	No
Behavioral consequence (e.g., nervous behavior)	No
Flock placement information	No
Heterogeneity	No
Feed transition	No
Routine treatment or prophylaxis (e.g., vaccination, routine supplementation)	No

Hereafter, the term baseline refers to the model-based reference range of expected locomotor behavior under routine conditions, as characterized by statistical modelling.

### Movement variables definition

Several variables were derived from the raw tracking data to quantify individual movement. The following notations were used:

• t denotes the frame number.
• $X_{i}\left(t\right)$ is the position vector of chicken i at time t, in image coordinates.
• $\Delta_{i}$ represents the time interval during which trajectory segment i appears in the image. The length of this interval, $|\Delta_{i}|$, corresponds to the observed duration for that trajectory segment.
• $V_{i}\left(t\right)=X_{i}\left(t+1\right)-X_{i}\left(t\right)$ denotes the velocity vector of trajectory segment i at time t, scaled by a constant factor (1/15). Its norm, $∥V_{i}\left(t\right)∥$, decreases as the animal's movement slows.
• $I_{t}$ is the set of trajectory-segment identifiers visible in the image at time t, and $n_{t}=\left|I_{t}\right|$ is the number of detected birds present at that moment.

Movement distances and velocities were expressed in units of chicken body length rather than in absolute physical units. This scaling provided a biologically interpretable measure of relative displacement and improved comparability across cameras despite minor residual differences in camera geometry.

Locomotor indicators were computed at the level of each 90-minute observation unit, defined by the combination of farm, flock, camera, sex, day of life, and recording period. Within each observation unit, all tracked trajectories available for the corresponding camera view and recording window were aggregated to compute the locomotor indicators. This structure preserved the camera-level sampling design while providing standardized observation units for subsequent indicator-specific analyses. For the modelling analyses, observations were retained from day 6 of life until the day of female removal, which occurred around day 32. The lower age limit was chosen to focus the analysis on the period in which trajectory-segment extraction was sufficiently stable for the computation of locomotor indicators. The upper limit was applied because rearing conditions changed substantially after female removal, particularly in the male area due to the increase in available space.

***Inactive Chickens.*** To quantify group-level inactivity, tracked trajectory segments were classified according to whether they showed sustained inactivity. Even when a bird is stationary, small apparent displacements may occur due to detection noise. To account for this, an adaptive velocity threshold was defined for each 90-min observation unit. Let S denote this threshold, computed as one-sixth of the median bounding-box diagonal in the corresponding observation unit, divided by the frame rate.

For each trajectory segment i, the proportion of time it remained below this threshold was computed as

$$p_{i}\left(S\right)=\frac{1}{\left|\Delta_{i}\right|}\sum_{t\in \Delta_{i}}1\left(∥V_{i}\left(t\right)∥\leq S\right),$$

where the indicator function 1(·) equals 1 when the condition is satisfied and 0 otherwise. A trajectory segment was classified as inactive if it remained below this movement threshold for more than 90 % of its observed duration. The 90 % cutoff was selected as an operational criterion to identify trajectory segments characterized by sustained inactivity, rather than derived through parameter optimization; the distribution of trajectory segments around this reference cutoff is shown in Supplementary Figure S4. All remaining trajectory segments, i.e., segments with more than 10 % of detections above the movement threshold, were classified as active. A descriptive sensitivity analysis was conducted using alternative cutoffs to evaluate how this choice affected trajectory-segment classification and segment-duration summaries (Supplementary Table S4 and Supplementary Figure S3).

Once trajectory segments were classified, the proportion of inactivity (PI) was computed for each group and each 90-minute observation unit as the proportion of retained trajectory segments classified as inactive. This segment-based proportion served as a group-level inactivity indicator and was used as the response variable in subsequent analyses. To assess the sensitivity of PI values to the cutoff choice, PI was recalculated under alternative cutoffs and compared with PI obtained using the reference 0.90 cutoff (Supplementary Table S5). For modelling purposes, a logit transformation was applied to PI to ensure approximate normality and compatibility with linear modelling, yielding the variable $PI_{logit}$.

A linear model including the main effects of day of life (continuous), day period (categorical: morning, afternoon, evening), sex (binary: female, male), and farm (categorical with four levels) was first fitted (Supplementary Table S6). Biologically plausible two-way interactions were then evaluated based on specific hypotheses: day x farm (whether inactivity trajectories differ between farms); sex x farm (whether sex effects vary across farms); farm x period (whether daily patterns differ by farm); day x sex (whether age-related changes differ between sexes); and period x sex (whether day period effects differ between sexes).

Each interaction was tested individually by comparing nested models using likelihood-ratio tests analysis of variance (ANOVA) and changes in Akaike Information Criterion (AIC). Interaction plots were also examined to assess biological interpretability. An interaction was retained if it significantly improved model fit (p < 0.050 and/or ΔAIC < −2) and induced a meaningful modification of the relationship (e.g., a change in slope or trajectory shape).

Based on these criteria, two interactions were retained: day × farm and day × sex. Although period × sex, period × farm and sex × farm interactions were statistically significant when tested individually, they were excluded to favor model parsimony, interpretability, and robustness to extrapolation. The final fixed-effects linear model is hereafter referred to as the *reduced model*. Observations from different flocks and cameras were not averaged before model fitting; each 90-min observation unit was retained as an individual observation. In the main reference model, camera and flock identifiers were retained as metadata but were not included as separate effects. This choice was made to avoid over-parameterization and to preserve model stability and interpretability.

Model performance was assessed using adjusted R², AIC, and residual diagnostics (normality, homoscedasticity, and influence analysis). To characterize the expected range of variability in $PI_{logit}$ under routine conditions, 95 % prediction intervals were derived from the reduced model. These intervals were subsequently used to identify deviations from expected locomotor baselines within an alert-based monitoring framework.

Model validation was conducted from complementary perspectives. First, internal predictive consistency was assessed using stratified and grouped cross-validation schemes reflecting different deployment scenarios, including generalization across flocks or farms. In stratified cross-validation, observations from all farms or flocks were represented across folds. In grouped cross-validation, all observations from a given flock or farm were held out together during validation, allowing model performance to be assessed on observational groups not used for model fitting. Second, the reduced model was applied, without refitting, to the non-routine dataset. This analysis examined whether observations assigned to the non-routine dataset were more likely to fall outside the model-based prediction intervals.

To further assess robustness across operational scenarios, three modelling strategies were compared: the reduced fixed-effects model, a mixed-effects model including farm as a random intercept, and a farm-agnostic model excluding farm and relying only on day of life, day period, sex, and their interaction with day. These strategies were compared in terms of goodness-of-fit, cross-validation performance under different schemes, and behavior when applied to the non-routine dataset.

***Active chickens****.* The activity of trajectory segments classified as active was summarized using the mean speed. For each track i, defined over its visible interval $\Delta_{i}$, the mean speed was computed as

$${\bar{v}}_{i}=\frac{1}{\left|\Delta_{i}\right|}\;\sum_{t\in \Delta_{i}}∥V_{i}\left(t\right)∥.$$

Because active trajectory segments have different observed durations $|\Delta_{i}|$, each ${\bar{v}}_{i}$ contributes a single value to the distribution of mean speeds within a given 90-min observation unit. The resulting window-level mean speed summarizes the movement intensity of active trajectory segments observed within the unit, rather than the mean speed of uniquely re-identified individuals. For a given 90-min observation unit w, the mean speed of active trajectory segments was defined as

$$Y_{w}=\frac{1}{N_{w}^{act}}\;\sum_{i\in T_{w}^{act}}{\bar{v}}_{i},$$

where $T_{w}^{act}$ is the set of active tracks and $N_{w}^{act}=\left|T_{w}^{act}\right|$. This scalar quantity $Y_{w}\;$serves as the response variable and summarizes movement intensity of active trajectory segments within the window.

The window-level mean speed of active birds $Y_{w}$ was modelled using a Gamma generalized linear model with a log link. This distributional choice applies only to the distribution of $Y_{w}$ across 90-min observation units, not to the underlying track-level values ${\bar{v}}_{i}$ or to the within-window distribution of trajectory-segment speeds. Let $\mu_{w}=E\left(Y_{w}|x_{w}\right)$ denote the conditional mean given predictors $x_{w}$ (day of life, day period, sex, farm). The linear predictor was specified as:

$$\log\left(\mu_{w}\right)=f_{day,\;period}\left(day_{w},\;period_{w}\right)+f_{sex,day}\left(day_{w}\right)sex_{w}+\beta_{farm}\left(farm_{w}\right),$$

where $f_{day,\;period}$ is a smooth interaction between day of life and day period, $f_{sex,day}$ is a sex-specific smooth effect of day, and $\beta_{farm}\;$are farm-specific intercepts. Smooth components were represented by cubic B-splines (three degrees of freedom), including spline-by-factor interactions. This structure allows for sex-specific temporal trajectories and diurnal variation, while absorbing systematic baseline differences between farms.

The choice of a Gamma distribution was motivated by the strictly positive and right-skewed nature of $Y_{w}$, together with empirical checks showing that variance scales approximately with the square of the mean. Under this parameterization, $V\left(Y_{w}|x_{w}\right)=\phi \mu_{w}^{2}$, with both $\mu_{w}$ and *ϕ* estimated from the data. For prediction, the implied Gamma distribution was parameterized as $\hat{k}=1/\hat{\phi }$, $\hat{\theta }=\hat{\mu_{w}}\hat{\phi }$.

Prediction intervals at the 95 % level were computed as

$$PI_{95\%}\left(w\right)\;=\;\left[q_{0.025}\left(\Gamma \left(\hat{k},\;\hat{\theta }\right)\right),\;q_{0.975}\left(\Gamma \left(\hat{k},\hat{\theta }\right)\right)\right],$$

where $q_{p}\left(\cdot \right)$ denotes the p-th quantiles of the Gamma distribution. Observations falling outside these limits were flagged as alerts, indicating deviations from the routine-condition reference range for the corresponding combination of day of life, day period, sex, and farm.

Alternative formulations (linear day effects, spline-based structures, fixed vs. mixed effects) were compared using AIC, diagnostic checks, and cross-validation. The Gamma model with sex-specific smooth day effects was selected as the main working model, hereafter referred to as the *Gamma_SexSmooth* model.

To examine model behavior outside the routine-condition domain used for baseline construction, the regression function and prediction-interval grid were applied, without refitting, to the non-routine dataset. The proportion of non-routine observations classified as alerts was summarized by farm and sex. This analysis provides insights into how often deviations from routine locomotor baselines occur under non-routine conditions, without implying specific welfare diagnoses.

***Collective Movements****.* To characterize collective movement (CM) events, an approach inspired by Vicsek and Zafeiris (2012) was applied to the individual velocity fields extracted from the tracking data.

At each time t, the average normalized velocity vector was computed as

$$\bar{V}\left(t\right)=\;\frac{1}{n_{t}V_{0}}\sum_{i\in I_{t}}V_{i}\left(t\right),$$

where $V_{0}=\frac{1}{n_{t}}\;\sum_{i\in I_{t}}∥V_{i}\left(t\right)∥$ is the mean speed at time t, and $n_{t}$ is the number of animals detected. Low values of $∥\bar{V}\left(t\right)∥$ indicate either slow movement or divergent directions among individuals, whereas values close to 1 reflect strong alignment of individual velocities, characteristic of collective motion.

Let $P\left(t\right)=\;∥\bar{V}\left(t\right)∥\in \left[0,1\right]$ denote this alignment signal over time. A CM was defined as a contiguous time interval $D=[t_{s},t_{e}]$ such that $P\left(t\right)>S_{m}$ for all t∈D, with $S_{m}$ an empirically determined threshold.

For each individual i, participation in a CM was quantified by the angular consistency between its instantaneous velocity vector and the group’s average direction. The proportion of time during which the alignment condition $|(V_{i}\left(t\right),\bar{V}\left(t\right))|\leq \pi /4$ was satisfied was computed as

$$d_{i}=\frac{\int_{T_{i}\cap D}1_{\left|\hat{V_{i}\left(t\right);\bar{V}\left(t\right)}\right|\leq \frac{\pi }{4}}dt}{|\;T_{i}\cap D|},$$

and an individual was considered to participate in the CM if $d_{i}\geq 0.8$.

Within each detected CM, three quantitative descriptors were derived from the alignment curve P(t):

• **Intensity** ($I_{CM}$): the peak alignment during the event, $I_{CM}=\max_{t\in D}P\left(t\right)$.
• **Area** ($A_{CM}$): the total magnitude of the collective movement, obtained as the area under P(t) curve,
  $$A_{CM}=\int_{t_{s}}^{t_{e}}P\left(t\right)dt,$$

which, for discrete frames sampled at frame rate f, corresponds to

$$A_{CM}=\frac{1}{f}\sum_{\tau =t_{s}}^{t_{e}}P\left(\tau \right).$$

• **Fraction involved** ($F_{CM}$): the proportion of animals identified as participants in the event.

Each CM event was linked to the 90-min observation unit in which it occurred and was assigned the corresponding farm, flock, camera, sex, day of life and day period, together with its event-level timing information. Two complementary analyses were conducted. The first was a descriptive analysis aimed at characterizing the frequency, magnitude, and participation of CM events under routine and non-routine recording conditions. Daily CM frequencies were summarized as mean ± 95 % confidence intervals across observation units defined by farm, flock, camera, sex, day of life and day period, and the distributions of CM structural descriptors ($A_{CM},\;I_{CM}$ and $F_{CM}$) were compared between the routine-condition training dataset and the non-routine dataset.

The second analysis focused on alert detection, designed to identify collective movements that were unusually large relative to the routine-condition reference distribution. For this purpose, CM area was normalized using a continuous-time baseline computed exclusively from the routine-condition training data, relying on the same temporal index previously defined for the modelling analysis to align recording periods across days.

For each farm x sex x temporal-index combination in the routine-condition training dataset, the reference CM area was defined as the median CM area. Each CM event was then normalized by the corresponding routine-condition median:

$$area_{rel}=\frac{A_{CM}}{{\tilde{A}}_{CM,ref}},$$

where ${\tilde{A}}_{CM,ref}$ is the routine-condition median CM area for the same farm x sex x temporal-index combination. Because the reference medians were computed from routine-condition events, routine-condition CM events had exact temporal reference values by construction. For non-routine CM events, an exact routine-condition reference was available only when the same farm × sex × temporal-index combination was represented in the training dataset. When no exact reference value was available, the closest available temporal index within the same farm × sex group was used. This nearest-reference procedure was required because CM events are discrete and routine-condition CM events were not necessarily observed for every temporal-index value needed to normalize events outside the routine-condition reference dataset.

Because the alert-based CM analysis focuses on unusually large collective movement events, only an upper threshold was defined. Alert thresholds were defined as the 95th percentile of $area_{rel}$ in the routine-condition dataset within each farm x sex x day period stratum. Events exceeding this threshold were labelled as alerts.

The proportion of alerts was then computed for both the routine-condition training dataset and the non-routine dataset, overall and by farm x sex, and compared using Fisher’s exact test with false discovery rate correction for multiple testing.

All analyses were performed using R 4.5.0. Model fitting used base R functions and the glmmTMB package, spline terms were constructed with splines, residual diagnostics were performed with DHARMa, and cross-validation routines were implemented using dplyr, tidyr, and purrr.

## Results

### Detection

The YOLOv8n model selected for the tracking pipeline showed high detection performance on the validation set, with precision = 0.95, recall = 0.92, and mAP50 = 0.96. This performance supported the subsequent tracking and locomotor-indicator extraction steps. Detailed comparisons with other YOLO variants are provided in Supplementary Table S16.

### Inactive chickens

The *reduced model* explained 80.7 % of the variance in logit-transformed inactivity (adjusted R² = 0.8072). Among the main effects, day of life was positively associated with inactivity (estimate = 0.0655, p < 0.001), indicating that inactivity progressively increased with age. Day period also significantly affected inactivity: compared to the morning, inactivity was higher during the afternoon (estimate = 0.2580, p < 0.001) and during the evening (estimate = 0.1623, p < 0.001). Sex had a significant effect, with males showing lower baseline inactivity than females (estimate = 0.1235, p < 0.001). Regarding farm effects, farm 2 (estimate = −0.4162, p < 0.001) and farm 4 (estimate = −0.3229, p < 0.001) showed significantly lower baseline inactivity than the reference farm (farm 1), whereas farm 3 did not differ significantly (estimate = 0.0833, p = 0.164).

Two interaction terms were retained in the final model. The day x farm interaction was highly significant (all contrasts p < 0.001), indicating that age-related inactivity trajectories differed between farms. The day x sex interaction was also significant (estimate = −0.0102, p < 0.001), showing that females exhibited a steeper increase in inactivity over time than males. These interaction effects are illustrated in Supplementary Figures S5 and S8.

Interactions involving day period x farm, day period x sex, and sex x farm were excluded. Although statistically significant when tested individually (Supplementary Table S7), visual inspection of the corresponding interaction plots (Supplementary Figures S6–S7 and S9) revealed that these terms mainly reflected differences in magnitude rather than qualitative changes in pattern (e.g., no reversals in direction or trajectory shape). To favor model parsimony, interpretability, and robustness to extrapolation, these interactions were omitted from the reduced model.

Detailed comparison with the full two-way interaction model is provided in Supplementary Tables S8–S9, and residual diagnostics for the reduced model are shown in Supplementary Figure S10 and described in Supplementary Section S3.3. Based on these analyses, the reduced model was retained for the construction of prediction intervals and alert evaluation. A descriptive sensitivity analysis showed that changing the trajectory-segment inactivity cutoff shifted the absolute level of PI, as expected, while PI values remained highly correlated with those obtained using the reference 0.90 cutoff (r = 0.978–0.999 across alternative cutoffs; Supplementary Tables S4–S5 and Supplementary Figure S3).

**Fig. 4:**
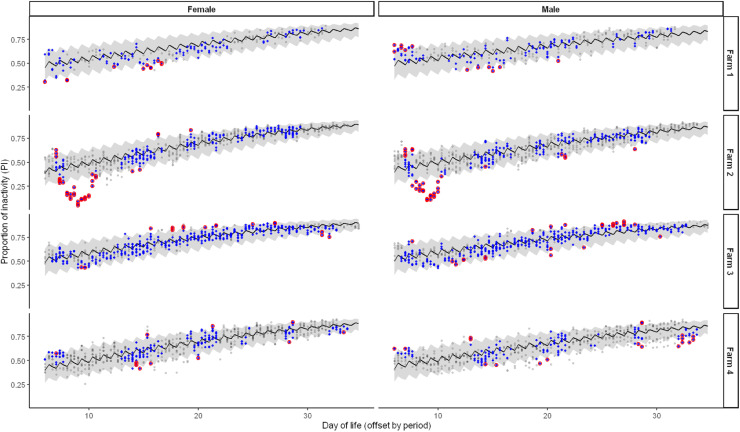
Proportion of inactivity with model-based 95 % prediction intervals and non-routine observations overlaid. Shaded bands represent the 95 % prediction intervals computed from the reduced regression model, and black lines show the corresponding model-predicted mean inactivity. Grey points represent observed inactivity values from the routine-condition training dataset, while blue points correspond to observations from the non-routine dataset. Red hollow circles indicate non-routine observations that fall outside the 95 % prediction intervals (alerts). The x-axis uses an offset transformation to jointly represent day of life and period of day within a single continuous scale. Panels display results by farm (rows) and sex (columns).

The 95 % prediction intervals defined the expected range of PI under routine-condition locomotor baselines. As expected from the construction of these intervals, approximately 5 % of observations from the routine-condition training dataset fell outside the predicted range. When the same regression function and prediction-interval grid were applied to the non-routine dataset, 9.5 % of observations fell outside the predicted range and were flagged as alerts (red hollow points in Fig. 4; Supplementary Table S11). This proportion indicates a greater frequency of deviations when observations fall outside the routine-condition domain used for baseline construction.

To assess robustness across modelling strategies, the reduced fixed-effects model was compared with two alternative formulations: a mixed-effects model including a random intercept for farm, and a farm-agnostic model excluding farm effects (Supplementary Table S10).

The mixed-effects model showed predictive performance comparable to the reduced model under stratified cross-validation schemes. Under both stratified-by-farm and stratified-by-flock designs, RMSE (root mean square error) and R^2^ values were similar to those of the reduced model (RMSE ≈ 0.301-0.302, R² ≈ 0.80). However, this formulation was associated with substantially higher AIC, and its marginal R^2^ was lower than that of the reduced model, while the conditional R^2^ was comparable, indicating that the random intercept accounted for limited additional variability. A mixed-effects specification including random slopes for day failed to converge and was therefore discarded.

The farm-agnostic model showed lower in-sample explanatory power than the reduced model (adjusted R^2^ = 0.788) and higher prediction error under stratified cross-validation schemes (RMSE ≈ 0.315-0.316). In contrast to the reduced model, the farm-agnostic formulation could be evaluated under grouped cross-validation by farm. In this setting, it outperformed the mixed-effects model, yielding lower RMSE and higher R^2^, indicating improved generalization when predicting inactivity patterns for farms not included during model training.

As a sensitivity analysis, alert rates obtained on the non-routine dataset were also computed for the mixed-effects and farm-agnostic formulations. All models yielded comparable proportions of alerts (9.0–10.3 %), indicating that alert detection was not strongly dependent on the specific model formulation (Supplementary Table S12).

### Active chickens

The Gamma_SexSmooth model provided a good overall fit and, most importantly, robust generalization under cross-validation schemes designed to mimic the operational scenario of predicting behavior in unseen flocks. The model achieved an AIC of −5682.6, with an estimated dispersion of σ² = 0.0598. In-sample predictive accuracy was high (RMSE = 0.129, mean absolute error (MAE) = 0.088, R² = 0.743).

Only one component of the spline-by-sex interaction reached statistical significance (z = 4.13, p < 0.001), indicating that sex-related differences in mean speed are driven by localized deviations in the smooth trajectory rather than by a global shift across age. Period effects were consistently negative relative to the morning reference, confirming lower movement intensity during the afternoon and evening and aligning with expected diurnal activity patterns.

Empirical mean–variance analyses and residual diagnostics supported the use of a Gamma distribution for modeling mean speed of active birds, with only mild departures from the ideal variance structure. Detailed results are provided in Supplementary Figures S11–S13.

Cross-validation confirmed stable predictive performance across scenarios. Under stratified-by-farm cross-validation, the mean RMSE was 0.130 (SD = 0.0058), and under stratified-by-flock cross-validation it was 0.131 (SD = 0.0036). In grouped-by-flock cross-validation, where entire flocks were excluded during training, the model maintained an RMSE of 0.144 (SD = 0.037). This level of performance was comparable to the best-performing alternative formulations while avoiding the loss of generalization observed for more complex farm-specific smooth structures (Supplementary Tables S13–S14). The selected model therefore balances flexibility, interpretability, and robustness to unseen data.

Analytic 95 % prediction intervals achieved an empirical coverage of 0.940, with 178 of 2,986 observations falling outside the nominal range. Coverage by farm was 0.944, 0.969, 0.926, and 0.924 for farms 1–4, respectively, and by sex was 0.931 (female) and 0.950 (male). These values remain close to the nominal 95 % level and indicate mild under-coverage in specific strata, consistent with slight dispersion heterogeneity detected in diagnostic analyses.

**Fig. 5:**
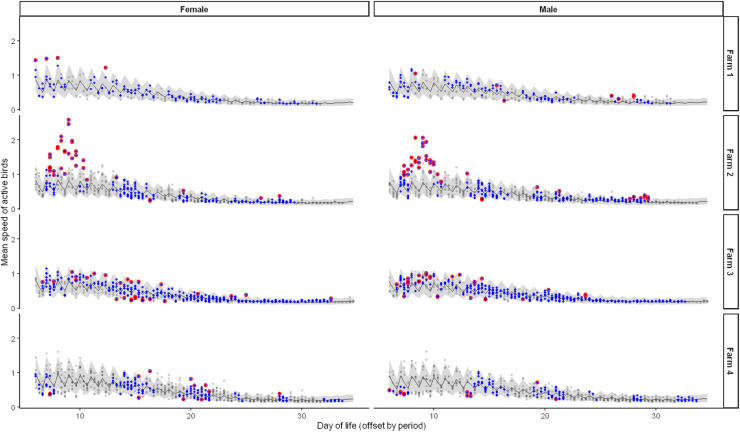
Fitted mean-speed trajectories and 95 % prediction intervals under the Gamma_SexSmooth model, with training and non-routine observations overlaid. Shaded ribbons represent the 95 % prediction intervals computed analytically from the Gamma model, and solid lines show the fitted mean-speed trajectories. Grey points correspond to observations from the routine-condition training dataset, while blue points represent observations from the non-routine dataset that fall within the prediction interval. Red hollow circles highlight non-routine observations outside the 95 % interval (alerts). The x-axis uses an offset transformation to jointly represent day of life and day period within a single continuous scale; the y-axis shows the window-level mean speed of active trajectory segments. Panels display results by farm (rows) and sex (columns).

Fig. 5 shows the fitted mean-speed curves and their associated 95 % prediction intervals generated by the Gamma_SexSmooth model, together with all available observations. Training data appear as grey points, while observations from the non-routine dataset are shown in blue. Non-routine observations falling outside the prediction interval and classified as alerts are highlighted as red hollow circles.

To examine model behavior outside the routine-condition domain used for baseline construction, the same regression function and prediction-interval grid were applied, without refitting, to the non-routine dataset (n = 1995; Fig. 5). A total of 184 observations (9.22 %) were identified as alerts, corresponding to mean speed values outside the expected range (Supplementary Table S15). Alert proportions varied across farms and sexes, ranging from 3.6 % (farm 1, females) to 17.3 % (farm 2, males). Most non-routine observations nonetheless remained within the prediction intervals, indicating that deviations from routine-condition locomotor baseline were concentrated in a limited subset of cases (Fig. 5).

### Collective movements

**Fig. 6:**
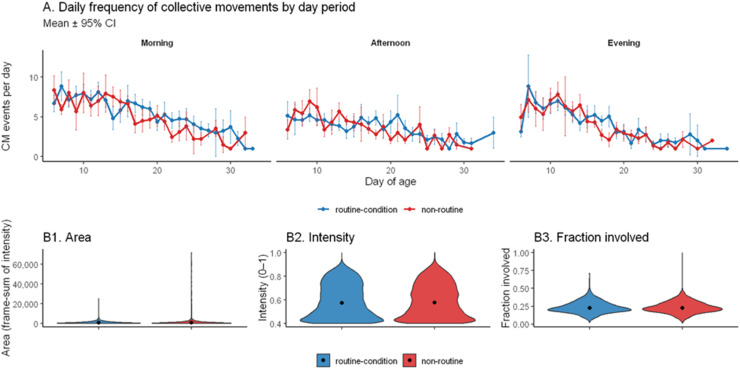
Descriptive characterization of collective movement (CM) events. **(A)** Daily frequency of CM events by age and recording period (morning, midday, evening), comparing the routine-condition training dataset and the non-routine dataset. Lines represent mean ± 95 % confidence intervals per day across all farms and sexes. **(B)** Distributions of CM structural descriptors—event area (integral of alignment intensity P(t) over event duration), peak intensity (maximum P(t)), and fraction of animals involved—comparing routine-condition and non-routine observations. Boxplots summarize medians, interquartile ranges, and 95 % ranges of values across all events.

The daily frequency of collective movement (CM) events showed a gradual decline with age across all recording periods (Fig. 6A). In the post-lights-on period, CM frequency was slightly higher at younger ages and decreased progressively as birds aged. Similar age-related declines were observed during the mid-light and pre-lights-off periods, indicating a consistent temporal pattern across daily light cycle. The routine-condition training dataset and the non-routine dataset displayed largely overlapping trajectories, with no systematic separation across age or recording period. This overlap suggests that daily CM frequency represents a recurrent feature of flock-level locomotor organization, rather than a descriptor that clearly separates routine-condition from non-routine observations in the descriptive analysis.

The structural characteristics of individual CM events were also comparable between datasets (Fig. 6B). The distributions of CM area, event intensity, and the fraction of animals involved showed substantial overlap between routine-condition and non-routine observations. Median values and upper-tail quantiles of area, intensity, and participation differed only marginally between datasets. These results indicate that the magnitude and collective participation of CM events are broadly conserved features of flock-level movement organization, even under non-routine recording conditions.

**Fig. 7:**
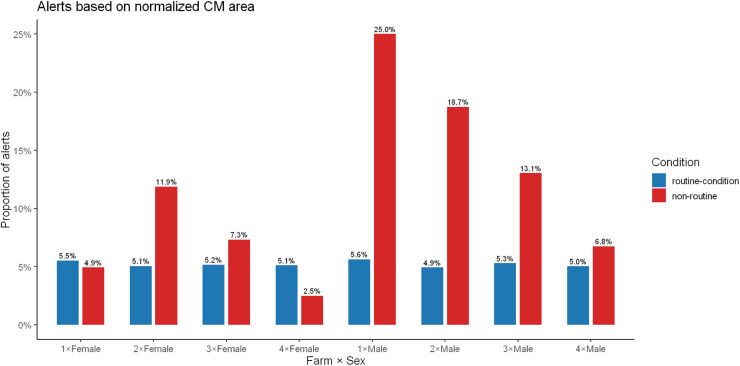
Alert-based comparison of collective movement events. Proportion of CM events exceeding the 95th percentile of normalized area (relative to routine-condition training medians) by farm × sex and dataset. Bars represent the proportion of alerts among scored events, with blue indicating the routine-condition training dataset and red indicating the non-routine dataset.

The continuous-time normalization procedure provided a reference value for all CM events included in the alert-based analysis. Across the 14243 CM events analyzed, 11936 were normalized using an exact temporal match, whereas 2307 required the nearest available reference within the same farm x sex group. For these nearest-reference cases, the median temporal distance was 0.33 days, the 90th percentile was 1 day, the 95th percentile was 1.33 days, and the maximum was 5.33 days. When CM events were analyzed using the relative, alert-based framework, clearer contrasts emerged (Fig. 7). After normalizing CM area by the training-only daily median on a continuous temporal index, a higher proportion of events from the non-routine dataset exceeded the 95th percentile of the routine-condition reference distribution. Overall, 5.1 % of CM events in the training dataset and 11.3 % in the non-routine dataset were classified as alerts (Fisher’s exact test$,\;p<2.2\;\times 10^{-16}$; odds ratio = 2.38, 95 % CI = 2.09–2.71).

Stratified analyses by farm × sex revealed consistent patterns for males, with all farms exhibiting higher alert rates in the non-routine dataset compared to the training data. In females, patterns were more heterogeneous: farms 2 and 3 showed increased alert rates under non-routine conditions, farm 1 showed similar rates across datasets, and farm 4 showed lower alert rates in the non-routine dataset. These results indicate that extreme collective movement events, when defined relative to routine-condition baselines, occur on average more frequently under non-routine conditions, with pronounced sex- and farm-dependent variability.

## Discussion

This study introduces a baseline-based framework for monitoring broiler chicken behavior using movement patterns extracted from video tracking in commercial production systems. Its main contribution is to define quantitative reference ranges for flock-level locomotor indicators under routine production conditions, against which new observations can be evaluated. By modelling the expected range of locomotor activity, this approach enables the identification of structured deviations in flock-level behavior across age, farms, and operational contexts. Rather than providing direct welfare diagnoses, alerts are interpreted as departures from routine-condition behavioral baselines that warrant contextual interpretation.

Within the scope of the present dataset, the proposed reference ranges are expected to be most transferable to commercial systems comparable to those studied here, namely farms using the same genetic line (Ross 308), a similar sex-segregated heavy broiler production system, and broadly similar recording and management conditions. Broader generalization to other genetic lines, housing designs, stocking-density trajectories, lighting programs, litter and climate conditions, health histories, or management practices would require additional validation, because these factors may alter both the expected level and temporal trajectory of locomotor indicators, as well as the residual variability used to construct prediction intervals.

### Detection, tracking, and trajectory-segment interpretation

The tracking component relied on a tracking-by-detection pipeline combining YOLOv8n and BoT-SORT, in which broilers were first detected in each frame and then associated across consecutive frames to generate trajectory segments. This approach provided a suitable balance between detection accuracy and computational cost for large-scale offline processing, and is consistent with recent detection-and-tracking strategies used to quantify broiler locomotion and mobility in poultry settings (Chen et al., 2023; Depuru, 2024; Jaihuni et al., 2023; Neethirajan, 2022; Siriani et al., 2022; Yang et al., 2024). Under commercial stocking densities, however, dense grouping, visual similarity among birds, occlusions, and exits from the camera field of view limit the possibility of maintaining persistent individual identities over long recording periods. This constraint is also recognized in recent work on video-based tracking of group-housed broilers, where trajectory fragmentation and interruptions are inherent challenges (Doornweerd et al., 2024).

Accordingly, the tracking output in the present study should be interpreted as trajectory segments rather than persistent individual identities across the full 90-min recording period. Intermittent detection could generate multiple trajectory segments corresponding to the same bird at different times, thereby affecting the relative contribution of birds with more fragmented trajectories. The duration summaries reported in Supplementary Section S2 should therefore be interpreted as durations of continuous trajectory segments, not as the time during which the same chicken remained visible or identifiable throughout the 90-min observation unit. These summaries provide information on the temporal continuity of the tracking output, rather than on individual residence time within the camera field of view.

### Event annotation quality and the operational definition of routine conditions

Event annotation in commercial production settings differs from event recording under controlled experimental conditions. In the present study, event logs were obtained from scheduled farm and veterinary monitoring and were typically documented at the day level or over multi-day intervals. Consequently, reported events could not always be precisely aligned with the 90-min video recordings, and the timing of any associated behavioral response could not be determined with certainty. This temporal imprecision, together with incomplete or heterogeneous reporting across farms, including variation in terminology and specificity, limits the extent to which deviations in locomotor indicators can be attributed to specific contextual events.

Both the routine-condition and non-routine datasets originated from the same observational data-generating process: they were collected from the same monitored flocks, within the same farms and rearing conditions, using the same camera system, recording schedule, and processing pipeline. The separation between the two datasets was applied after data collection, based on farm and veterinary records rather than on the movement indicators themselves. Because event records may be incomplete, the routine-condition dataset cannot be assumed to be entirely free of unreported events. It should therefore not be interpreted as representing optimal welfare or the absence of all stressors, but as an empirical behavioral reference reflecting stable commercial functioning in the absence of reported perturbations. Conversely, the non-routine dataset should be interpreted as a pragmatic set of observations enriched in reported non-routine conditions, rather than as exhaustive ground truth for confirmed welfare or health impairments.

Because manual review of more than 14,000 hours of video footage was not feasible, event records provided the available contextual reference for constructing and evaluating routine-condition baselines. Higher-resolution event annotation and contextual data would be needed for more specific attribution of observed behavioral deviations.

### Locomotor indicators, baseline models, and biological interpretation

Building on the trajectory-segment structure of the tracking output, the locomotor indicators were constructed by aggregating short-term movement information within each observation window, without requiring persistent individual identities across the full recording period. The three indicators analyzed in this study were evaluated separately but capture complementary dimensions of flock locomotor organization. The proportion of inactivity summarized sustained low-movement trajectory segments and relates to the broader use of group-level inactivity or activity descriptors as behavioral proxies in automated welfare surveillance frameworks (Aydin, 2017; Dawkins et al., 2012). Mean speed of active trajectory segments reflects movement intensity among segments not classified as inactive, while collective movement events capture transient episodes of directional alignment within the flock.

This aggregation strategy is consistent with the broader logic of automated flock-level behavioral monitoring, where group-level motion descriptors can be informative even without persistent long-term individual identification. Optical-flow-based approaches have similarly shown that aggregate motion patterns can support contextual interpretation of broiler flock behavior and provide early warning signals for health- or welfare-relevant deviations (Colles et al., 2016; Dawkins et al., 2009, 2012, 2017; Gebhardt-Henrich et al., 2021; van der Eijk et al., 2022). However, the present trajectory-based framework differs from optical flow by deriving interpretable locomotor descriptors from trajectory segments before aggregation, allowing inactivity, movement intensity, and collective coordination to be evaluated as distinct components of routine-condition flock dynamics. More broadly, alterations in locomotor patterns have been associated with lameness, footpad dermatitis, thermal discomfort, and other welfare-relevant challenges (Aydin et al., 2010; Dawkins et al., 2017), supporting the relevance of these descriptors for deviation-based surveillance, while not implying direct welfare diagnosis.

### Inactivity model

The statistical modelling of inactivity revealed structured effects of age, day period, sex, and farm. Inactivity increased with age, likely reflecting natural changes in mobility and behavioral organization as birds grow heavier, consistent with previous reports of increased resting time and reduced walking or exploratory behaviors in older broilers (Bessei, 2006; Bokkers and Koene, 2003; Giersberg et al., 2023; Trocino et al., 2020; Weeks et al., 2000). Day period also shaped inactivity patterns, with higher inactivity during the mid-light and pre-lights-off periods relative to the post-lights-on period. This pattern is consistent with diurnal modulation of broiler behavior, shaped by light schedules, ambient conditions, and daily management routines (Blatchford et al., 2009), and with previous reports of higher activity earlier in the light period followed by a gradual decline later in the daily light cycle (Guo et al., 2022). Sex differences were also evident, with females generally more inactive than males, in line with previous studies reporting higher locomotor activity and broader gait repertoires in male broilers (Bokkers and Koene, 2002; Rose et al., 2015; Trocino et al., 2020).

Two interactions were retained in the final inactivity model. The day × sex interaction indicates that the age-related increase in inactivity differed between males and females, but this pattern should not be interpreted as a general sex-specific aging trajectory. Because body weight was not included as an explicit covariate, this interaction may partly reflect differences in growth rate and effective live-weight stocking density between sexes, even when density in birds/m² was similar before female departure. Future analyses combining video-derived locomotor indicators with repeated body-weight measurements would be needed to disentangle the effects of chronological age, sex, growth, and stocking density on inactivity patterns.

The day × farm interaction highlighted that the rate at which inactivity increased with age differed across farms. In addition, although the period × farm interaction was not retained in the final reference model, it showed farm-specific differences during the pre-lights-off period, with inactivity remaining high in some farms but decreasing in others. Together, these patterns suggest that between-farm heterogeneity affected both age-related inactivity trajectories and the expression of inactivity at specific times of the daily light cycle. Such differences may reflect farm-specific environmental, management, and production-related conditions, including litter quality, microclimate, ventilation, lighting programs, end-of-day management routines (Dawkins et al., 2004), as well as flock-specific production histories, growth trajectories, and effective live-weight stocking-density trajectories. Because these factors were not explicitly incorporated in the present analysis, their contribution to farm-specific inactivity patterns could not be disentangled.

Other statistically significant interactions were excluded to favor parsimony and robustness. The period × sex interaction was not retained because sex-related differences persisted across periods with only limited variation in magnitude. Similarly, the sex × farm interaction reflected differences in effect magnitude rather than direction, with females consistently more inactive than males across farms. These interactions were therefore considered less informative for defining a robust routine-condition reference model than the retained day × sex and day × farm effects.

Production flock may also capture variability related to flock-specific conditions, management, and calendar time; however, with only three consecutive flocks per farm and non-synchronized production cycles across farms, flock could not be interpreted as a direct proxy for season. Variation across flocks was therefore considered during model evaluation through cross-validation schemes, while unmodelled environmental, flock-, or season-related variability should be considered when interpreting between-farm differences and model generalization.

A key methodological question addressed in this study concerns generalization to new farms. The reduced fixed-effects model achieved strong explanatory power but explicitly incorporated farm-specific effects. Alternative modelling strategies therefore offer complementary insights. The mixed-effects model with a random intercept for farm achieved comparable performance under stratified cross-validation, but did not systematically outperform the reduced model and was associated with higher information criteria. More complex random-slope specifications proved unstable, likely reflecting the limited number of farms, consistent with known challenges in random-effects estimation under sparse grouping structures (see e.g., McNeish and Stapleton, 2016).

In contrast, although the farm-agnostic model had lower explanatory power on the training data, its performance under grouped cross-validation, particularly when entire farms were held out, was comparable to, and in some cases better than, the other formulations evaluated. These results indicate that modelling choices should be guided by the intended deployment context: farm-specific models may be preferable when calibration data are available, whereas farm-agnostic models may provide a viable alternative for screening or initial deployment in previously unseen farms.

### Mean-speed model

The Gamma-SexSmooth model captured the temporal evolution of mean speed in active trajectory segments, reproducing sex- and period-specific differences in daily locomotor rhythms. Its predictive performance remained stable across farms and flocks, supporting its use within baseline-based behavioral surveillance frameworks under commercial conditions.

Consistent with established knowledge of broiler behavior, the model reproduced the expected daily rhythm, with higher movement intensity during the post-lights-on period, reduced movement during the mid-light period, and the lowest values during the pre-lights-off period. This pattern likely reflects a combination of lighting schedules, feeding routines, and progressive spatial constraint as birds grow and occupy a larger proportion of the available floor area. Although farm effects were statistically significant, they did not substantially alter the overall speed trajectories, suggesting that a single calibrated reference model can generalize across farms operating under comparable production systems. Nonetheless, farm-specific calibration over longer time horizons may further refine reference ranges and improve adaptation to local conditions.

Mean speed summarizes the central tendency of movement intensity among active trajectory segments, but it may not capture all forms of locomotor change. In some cases, deviations may involve changes in the distribution of trajectory-segment speeds, such as increased skewness or multimodality when only a subset of segments is affected, while the window-level mean remains largely unchanged. Although these distribution-level analyses were not formalized in the present study, they may provide useful complementary descriptors for future behavioral surveillance frameworks.

### Collective movements as a baseline-based behavioral descriptor

The collective movement (CM) analysis extends the characterization of flock-level locomotor behavior beyond mean-speed summaries by focusing on transient episodes of strong directional alignment among detected velocity vectors. This approach is conceptually grounded in models of collective motion in biological systems (Vicsek and Zafeiris, 2012). Whereas the Gamma-based speed model captures gradual, age-dependent changes in average movement intensity, the CM framework isolates discrete episodes of coordinated motion. Together, these approaches provide complementary views of flock mobility: one describing continuous locomotor dynamics, the other highlighting episodic synchronization.

A first descriptive analysis showed that CM frequency decreased steadily with age across all recording periods, consistent with the well-documented reduction in overall locomotor activity as broilers grow. Although reduced space may facilitate disturbance propagation, whereby movement by one bird triggers responses in nearby birds, the overall decline in CM frequency with age suggests that reduced mobility predominated over any such effect. The structural characteristics of CM events, summarized by their area, peak alignment intensity, and the fraction of detected trajectories involved, displayed highly similar distributions across the routine-condition training dataset and the non-routine dataset. This stability indicates that coordinated group motion is a common and recurrent feature of broiler flock organization, rather than an exceptional or pathological phenomenon. Comparable observations have been reported in studies using optical-flow-based and related computer-vision metrics to quantify flock-level movement organization (Dawkins et al., 2009, 2012; Gebhardt-Henrich et al., 2021; Pereira et al., 2021).

However, similarity in descriptive distributions does not imply equivalence across behavioral contexts. When CM magnitude was reframed relative to routine-condition baselines, defined by farm, sex, and day period on a continuous temporal scale, clear differences emerged in the upper tails of the distribution. Events exceeding the 95th percentile of their normalized routine-condition reference were approximately twice as frequent in the non-routine dataset. Thus, what distinguished non-routine contexts was not the presence of collective motion per se, but the increased occurrence of events that were unusually large or intense relative to the expected pattern for a given age, period, sex, and farm context. The effect was consistent across farms in male areas, whereas more heterogeneous patterns were observed in female areas, suggesting sex- and context-dependent baselines that may reflect differences in growth trajectories, effective stocking density, spatial organization, or local environmental conditions.

Interpreting elevated CM intensity requires caution. High-alignment and large-area collective movements do not necessarily reflect panic, distress, or locomotor impairment; they may also arise from vigorous coordinated locomotion, adaptive collective reorganization, transient crowding responses, abrupt external stimuli, or short-lived disruptions such as human entry, noise, or management interventions. This interpretation is consistent with previous work linking video-derived flock motion signals to external perturbations and health-related challenges (Colles et al., 2016). As emphasized by Dawkins (2024), the capacity to move rapidly and coherently can itself reflect adequate musculoskeletal function and environmental support. Distinguishing adaptive collective activity from potentially problematic mobilization would therefore require richer, higher-resolution contextual annotation and, ultimately, supervised modelling approaches trained to classify collective movement patterns. Accordingly, the present framework interprets collective movement intensity in relation to routine-condition references, without assuming its underlying causes.

Finally, defining collective movement using the norm of the average normalized velocity vector assumes a unimodal distribution of movement directions. Under this assumption, strong alignment corresponds to coordinated group motion. Situations in which multiple subgroups move simultaneously in opposing directions would not be captured by this definition, although such cases could in principle be detected by analyzing multimodality in the distribution of movement directions. Exploring such extensions represents a natural avenue for future methodological refinement.

### Deviation-based monitoring across indicators

Prediction intervals derived from routine-condition baseline models defined the expected range of flock-level locomotor indicators under routine production conditions. Observations falling outside these intervals were therefore interpreted as model-defined alerts, that is, departures from the expected routine-condition range. When applied to the non-routine dataset, alerts were more frequent than the nominal 5 % expected under the routine-condition reference, reaching approximately 9.5 % for inactivity, 9.2 % for mean speed, and 11.3 % for normalized collective movement magnitude. Thus, all three indicators captured departures from baseline more often in the non-routine dataset, although most non-routine observations remained within the expected range.

This modest increase indicates that reported non-routine conditions were associated with detectable changes in some dimensions of flock locomotor organization, but only for a subset of observations. It also raises important questions for future work regarding which types of non-routine events are likely to generate detectable locomotor deviations, which indicators are most sensitive to specific contexts, and which changes require additional behavioral, environmental, or health-related information. Departures may occur in one indicator, in several indicators simultaneously, or may not be captured by the indicators considered here. Their detectability is inherently event-dependent, and uncertainty in the timing and resolution of recorded events further constrains causal interpretation.

A conceptually related approach was proposed by Chen et al. (2023), who combined video-based tracking with autoregressive time-series modelling for anomaly detection in broiler flocks. Such approaches can capture complex temporal dependencies in movement patterns and are closely aligned with the objective of identifying departures from expected flock behavior. The present framework differs mainly in how the behavioral reference is defined: reference ranges were constructed from observations selected a priori to represent routine production conditions, whereas anomaly-detection approaches may incorporate atypical observations into the learned reference if no explicit distinction is made between routine and non-routine conditions. This distinction makes the construction of the baseline more transparent, while the interpretation of deviations remains dependent on contextual information.

### Perspectives

The integration of video tracking and artificial intelligence offers a framework for monitoring broiler locomotor behavior under commercial production conditions. The present results support the use of routine-condition behavioral baselines to identify deviations in flock-level locomotor organization across farms, ages, and operational contexts. However, the current framework should be viewed as a methodological step toward behavioral surveillance, rather than as a ready-to-use on-farm decision system.

Future work should primarily address the interpretation and operational validation of deviations generated by baseline-based monitoring systems. In the present study, event information was available mainly as coarse farm-level records, which allowed the construction of a routine-condition reference dataset but did not support causal attribution of individual alerts to specific events. A key next step will therefore be to prospectively collect higher-resolution contextual annotations, including the timing, duration, severity, and type of management, environmental, or health-related events, and to systematically review observations falling outside prediction intervals against these records. Such validation would help determine which alerts correspond to operationally relevant changes, which reflect transient fluctuations, and which cannot be interpreted without additional indicators beyond locomotor behavior.

The present study analyzed inactivity, mean speed, and collective movement as separate but complementary indicators. A future step will be to integrate these descriptors within a formal multivariate framework, allowing deviations to be evaluated jointly rather than through independent indicator-specific thresholds. Such an approach could help determine whether concurrent, weak, or discordant changes across indicators provide more informative screening signals than any single descriptor alone. This integration could be strengthened by incorporating environmental and production data, adaptive baseline calibration, and farm-specific validation procedures. Future implementations should also evaluate camera placement and coverage, including whether broader spatial sampling or complementary viewing angles add useful behavioral information under dense commercial conditions. At larger deployment scales, privacy-preserving collaborative modelling approaches, such as federated learning, may become relevant when developing shared reference models across farms without requiring centralized access to sensitive farm-level data (Wang et al., 2023; Wu et al., 2022).

### Animal use

The study was conducted under routine commercial production conditions in broiler farms. No experimental procedures, invasive interventions, or modifications of standard management practices were performed for the purpose of this research. All animals were managed according to standard commercial husbandry practices in compliance with applicable European and national regulations on animal welfare. As the study relied exclusively on non-invasive video recordings collected under routine farm conditions, no specific ethical approval was required.

## CRediT authorship contribution statement

**Noslen Hernández:** Writing – review & editing, Writing – original draft, Visualization, Methodology, Formal analysis, Data curation, Conceptualization. **Sylvain L’Hermite:** Writing – review & editing, Software, Methodology, Data curation. **Pauline Créach:** Writing – review & editing, Validation, Project administration, Investigation. **Didier Concordet:** Writing – review & editing, Supervision, Methodology, Conceptualization.

## Disclosures

The authors declare that they have no known competing financial interests or personal relationships that could have appeared to influence the work reported in this paper.

## Appendix Supplementary materials

Image, application 1

## Footnotes

Supplementary material associated with this article can be found, in the online version, at doi:10.1016/j.psj.2026.107363.
