# Supplementary material for: Characterizing locomotor behavior variability in commercial broiler flocks using large-scale video tracking
Source: Poult Sci. 2026 Jun 30;105(10):107363. doi: 10.1016/j.psj.2026.107363 (PMC13352383; doi:10.1016/j.psj.2026.107363)
Supplement: Supplementary file 1 [file mmc1.docx]

**Characterizing locomotor behavior variability in commercial broiler flocks using large-scale video tracking**

Noslen Hernández^1,*^**,** Sylvain L’Hermite^1^ , Pauline Créach^3^, Didier Concordet^1,2^

^1^Université de Toulouse, INRAE, UMR InTheres, Toulouse, France

^2^école Nationale Vétérinaire de Toulouse, Toulouse, France

^3^ITAVI, 7 rue du Faubourg Poissonnière, 75009 Paris

* corresponding author

**(Supplementary Material)**

**S1. Event logs and construction of the non-routine dataset**

| **Event category** | **Description** | **Source of information** |
| --- | --- | --- |
| **Technical and management events** | | |
| Accès Assiettes | Feed-pan access issue. | Farm technical/feed-management records. |
| Accès Pipettes | Drinker/nipple access issue. | Farm technical/water-system records. |
| AJOUT GRIT | Grit addition. | Farm feeding-management records. |
| Ajout litière | Litter addition. | Farm management records. |
| Ajout turbines | Addition or activation of turbines. | Farm technical/environmental records. |
| Problème technique aliment | Feed-system technical issue. | Farm technical/feed-system records. |
| Problème technique eau | Water-system technical issue. | Farm technical/water-system records. |
| Problème technique ventilation | Ventilation technical issue. | Farm technical/environmental records. |
| Réglage abreuvement | Drinker adjustment. | Farm water-system management records. |
| Réglages ventilation | Ventilation adjustment. | Farm environmental-management records. |
| Mise à jeun | Feed withdrawal or fasting. | Farm feeding-management records. |
| **Health-related events** | | |
| Boîteries | Reported lameness or gait abnormality. | Farm or veterinary health monitoring. |
| Coccidiose | Suspected or reported coccidiosis. | Farm and veterinary health monitoring. |
| Colibacillose | Suspected or reported colibacillosis. | Farm and veterinary health monitoring. |
| Digestif | Suspected digestive disorder. | Farm or veterinary health monitoring. |
| Entérocoques | Suspected or reported enterococcal issue. | Farm or veterinary health monitoring. |
| Mortalité | Mortality event. | Farm mortality or veterinary records. |
| Hypoglycémie | Suspected hypoglycemia. | Farm or veterinary health monitoring. |
| Halètement | Panting. | Farm or veterinary observations. |
| **Environmental event** | | |
| Hygrométrie | Abnormal humidity record. | Farm environmental records. |
| Mauvaise ambiance | Poor indoor environment. | Farm environmental monitoring. |
| NH3 | Abnormal ammonia record. | Farm environmental records. |
| Poussière | Dust issue. | Farm environmental records. |
| Taux CO2 | Abnormal CO₂ record. | Farm environmental records. |
| Température extérieure | Outdoor-temperature-related event. | Farm environmental records. |
| Température intérieure | Indoor-temperature-related event. | Farm environmental records. |
| Vitesse d'air | Abnormal air-speed record. | Farm environmental records. |
| Coup de chaleur (mortalité élevée) | Heat-stress episode with increased mortality. | Farm environmental or veterinary records. |
| **Human presence, handling, and sampling events** | | |
| Chiffonnettes | Environmental or regulatory sampling. | Farm, veterinary, or regulatory monitoring records. |
| Maintenance extérieure | External maintenance work. | Farm technical or maintenance records. |
| Passage éleveur | Farmer entry or passage. | Farm management records. |
| Pesées manuelles | Manual weighing. | Farm or veterinary management records. |
| Taguage poulets | Bird tagging or marking. | Farm or veterinary handling records. |
| Visite | Technical, veterinary, or project visit. | Visit or monitoring records. |
| **Production-management events** | | |
| Desserrage | Thinning or partial bird removal. | Farm production-management records. |
| Enlèvement | Bird removal or depopulation event. | Farm production-management records. |
| Fermeture volets | Closure of shutters. | Farm environmental or light-management records. |
| **Feeding or behavioral observations** | | |
| Compétition alimentaire | Feeding competition. | Farm behavioral or feeding observations. |
| Baisse de consommation eau | Reduced water consumption. | Production and water-consumption records. |
| Tri alimentaire | Feed sorting or feed-selection issue. | Farm feeding observations. |

**Table S1. Event categories included in the non-routine dataset. Event category names are reported as recorded in the original farm event database; English descriptions are provided for clarity. Categories are grouped thematically to improve readability. The table summarizes the main types of contextual events used to define observations under non-routine conditions. These records were obtained from farm technical logs, production records, routine farm observations, and veterinary monitoring. Health-related categories correspond to suspected or reported events recorded during routine monitoring and should not be interpreted as systematically confirmed diagnoses.**

**S2. Tracking output and trajectory-segment duration**

| **Farm** | **Segment category** | **N trajectory segments** | **Median duration** | **Mean duration** | $\mathbf{Q1-Q3}$ | **P95** | **Min-Max** |
| --- | --- | --- | --- | --- | --- | --- | --- |
| All farms | All retained | 126,643,977 | 0.67 | 2.26 | 0.20-2.29 | 9.96 | 0.03–94.88 |
| All farms | Active | 44,848,395 | 0.37 | 0.81 | 0.15–0.95 | 3.06 | 0.03–62.91 |
| All farms | Inactive | 81,795,582 | 1.06 | 3.06 | 0.27–3.55 | 12.80 | 0.03–94.88 |
| Farm 1 | All retained | 28,444,901 | 0.63 | 2.01 | 0.21–2.05 | 8.60 | 0.03–94.25 |
| Farm 1 | Active | 10,556,028 | 0.37 | 0.79 | 0.16–0.93 | 2.94 | 0.03–62.91 |
| Farm 1 | Inactive | 17,888,873 | 0.98 | 2.73 | 0.27–3.16 | 11.17 | 0.03–94.25 |
| Farm 2 | All retained | 31,173,376 | 0.62 | 2.34 | 0.19–2.26 | 10.65 | 0.03–91.15 |
| Farm 2 | Active | 12,266,088 | 0.34 | 0.77 | 0.14–0.90 | 2.97 | 0.03–39.19 |
| Farm 2 | Inactive | 18,907,288 | 1.09 | 3.35 | 0.26–3.91 | 14.27 | 0.03–91.15 |
| Farm 3 | All retained | 28,287,789 | 0.73 | 2.53 | 0.21–2.64 | 11.26 | 0.03–94.80 |
| Farm 3 | Active | 8,509,841 | 0.37 | 0.82 | 0.15–0.97 | 3.08 | 0.03–44.28 |
| Farm 3 | Inactive | 19,777,948 | 1.11 | 3.26 | 0.27–3.89 | 13.68 | 0.03–94.80 |
| Farm 4 | All retained | 38,737,911 | 0.70 | 2.20 | 0.21–2.26 | 9.40 | 0.03–94.88 |
| Farm 4 | Active | 13,516,438 | 0.38 | 0.85 | 0.15–1.00 | 3.21 | 0.03–46.69 |
| Farm 4 | Inactive | 25,221,473 | 1.06 | 2.93 | 0.28–3.34 | 12.06 | 0.03–94.88 |

**Table S2.** **Duration of retained trajectory segments used for locomotor-indicator computation. Durations are summarized globally and by farm for all retained trajectory segments and separately for segments classified as active or inactive.** Summaries were computed over the analysis period extending from day 6 of life to the day of female removal from the house**. Segments with fewer than 30 detections were excluded, corresponding to a minimum observed duration of 2 s at 15 frames per second. Segment classification was based on the 90% inactivity cutoff described in the Methods. N denotes the number of retained trajectory segments; Median and Mean describe the central tendency of segment duration; Q1–Q3 denotes the interquartile range; P95 denotes the 95th percentile; and Min–Max gives the observed range. Duration values are reported in minutes.**

| **Farm** | **Segment category** | **N units** | **Median** | $\mathbf{Q1-Q3}$ | **P5-P95** |
| --- | --- | --- | --- | --- | --- |
| All farms | All retained | 5,663 | 21,224 | 15,065.5–28,106.0 | 6,994.4–40,558.3 |
| All farms | Active | 5,663 | 6,178 | 3,165.0–11,091.5 | 877.0–19,932.4 |
| All farms | Inactive | 5,663 | 14,158 | 10,585.5–17,925.0 | 5,424.5–23,608.4 |
| Farm 1 | All retained | 1,005 | 27,504 | 21,087.0–34,348.0 | 7,730.2–48,219.0 |
| Farm 1 | Active | 1,005 | 9,502 | 5,047.0–14,087.0 | 1,208.4–23,572.6 |
| Farm 1 | Inactive | 1,005 | 18,124 | 14,351.0–21,432.0 | 6,426.4–26,370.4 |
| Farm 2 | All retained | 1,529 | 19,357 | 14,849.0–24,907.0 | 6,414.4–35,396.8 |
| Farm 2 | Active | 1,529 | 5,608 | 3,006.0–11,083.0 | 869.8–20,606.0 |
| Farm 2 | Inactive | 1,529 | 12,258 | 10,209.0–15,015.0 | 5,066.2–18,600.6 |
| Farm 3 | All retained | 1,537 | 18,500 | 13,747.0–22,647.0 | 6,616.4–29,618.4 |
| Farm 3 | Active | 1,537 | 4,514 | 2,528.0–7,423.0 | 856.6–13,399.6 |
| Farm 3 | Inactive | 1,537 | 12,921 | 10,367.0–15,649.0 | 5,421.4–19,258.4 |
| Farm 4 | All retained | 1,592 | 25,046 | 14,307.8–31,281.5 | 7,752.6–43,167.7 |
| Farm 4 | Active | 1,592 | 7,006.5 | 3,475.8–12,162.5 | 847.6–20,775.1 |
| Farm 4 | Inactive | 1,592 | 16,190.5 | 9,896.2–20,528.8 | 5,440.7–24,924.8 |

**Table S3.** **Number of retained trajectory segments per 90-min observation unit. Counts are summarized globally and by farm for all retained trajectory segments and separately for active and inactive trajectory segments.** Summaries were computed over the analysis period extending from day 6 of life to the day of female removal from the house**. Segments with fewer than 30 detections were excluded, corresponding to a minimum observed duration of 2 s at 15 frames per second. N units denotes the number of 90-min observation units; Median denotes the median number of retained trajectory segments per observation unit; Q1–Q3 denotes the interquartile range; and P5–P95 denotes the 5th to 95th percentile range.**


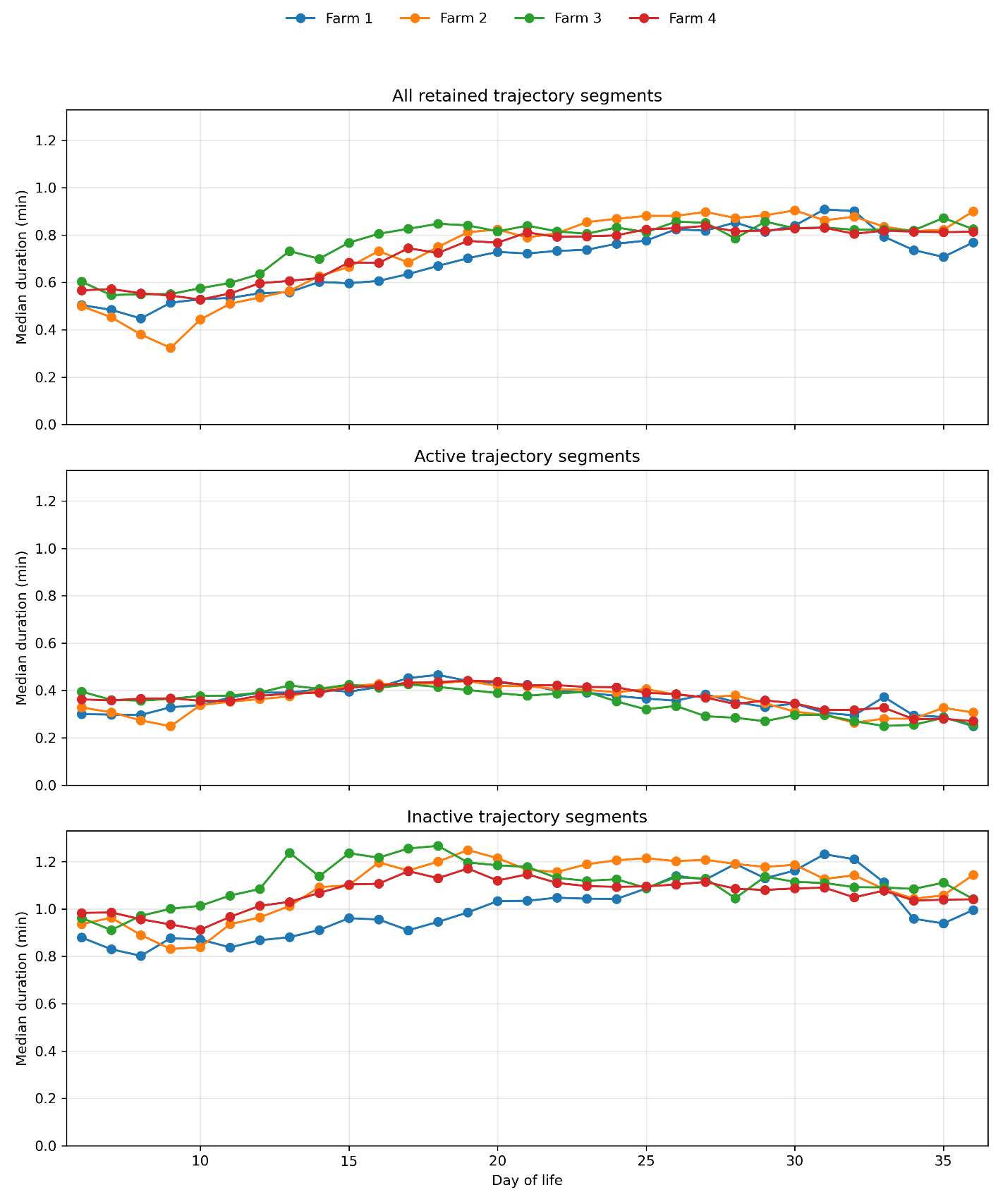


**Figure S1. Daily median duration of retained trajectory segments by farm and day of life. The figure shows the median duration of retained trajectory segments during the analysis period** extending from day 6 of life to the day of female removal from the house**. Lines are shown separately by farm and by segment type: all retained trajectory segments, active trajectory segments, and inactive trajectory segments. Segments with fewer than 30 detections were excluded, corresponding to a minimum observed duration of 2 s at 15 frames per second. Active and inactive labels refer to the trajectory-segment classification used for locomotor-indicator computation.**


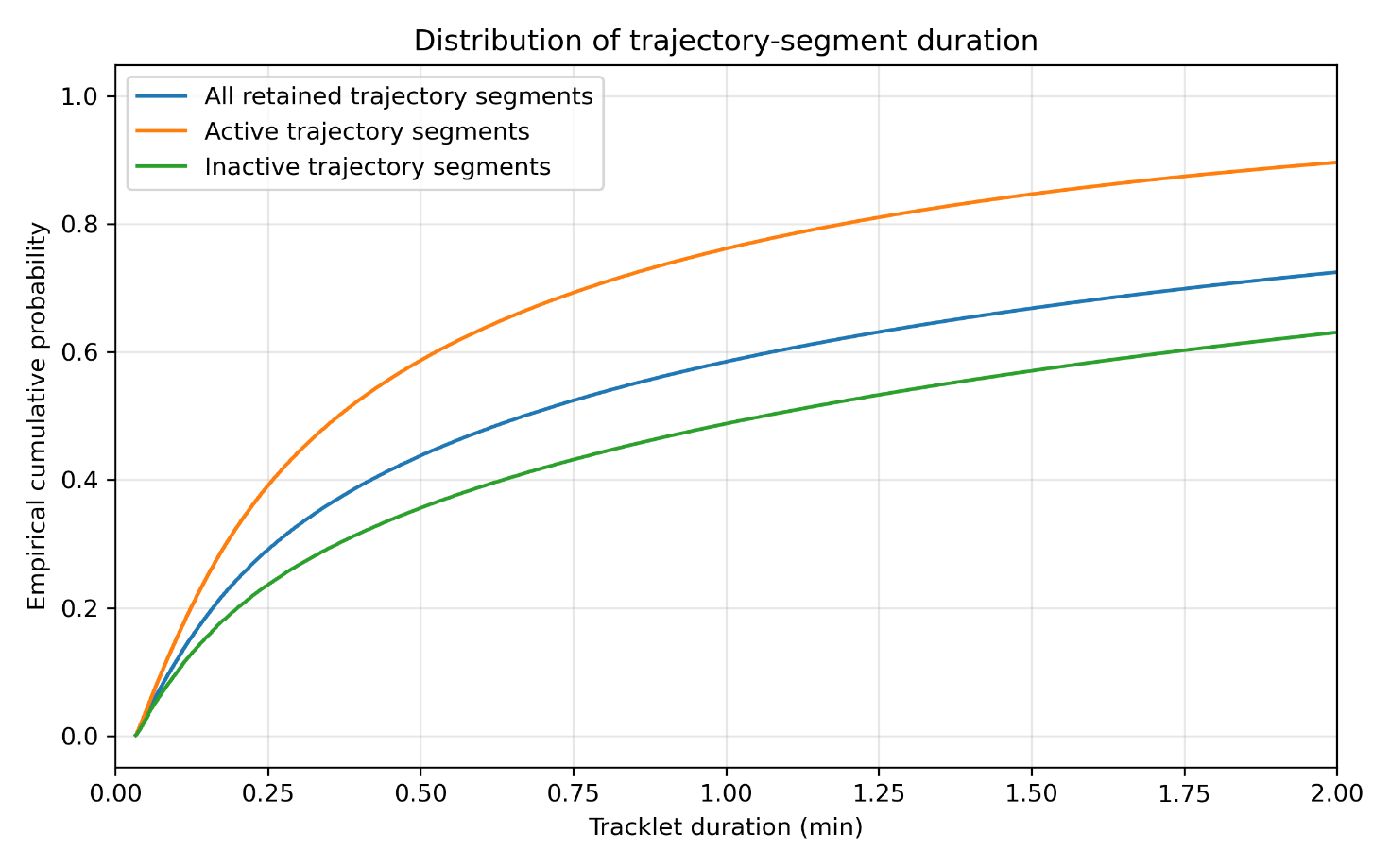


**Figure S2. Empirical cumulative distribution of retained trajectory-segment duration. Curves show the cumulative distribution of duration for all retained trajectory segments, active trajectory segments, and inactive trajectory segments during the analysis period** extending from day 6 of life to the day of female removal from the house**. The x-axis is truncated to the first 2 min to emphasize the range containing most short trajectory segments. Segments with fewer than 30 detections were excluded, corresponding to a minimum observed duration of 2 s at 15 frames per second. Full distribution summaries, including upper-tail percentiles and maximum values, are provided in Table S2.**

**S3. Inactive chickens**

**S3.1 Sensitivity to the inactivity cutoff**

| **Cutoff** | **Active (%)** | **Inactive (%)** | **Median duration: all** | **Median duration: active** | **Median duration: inactive** |
| --- | --- | --- | --- | --- | --- |
| 0.80 | 21.6 | 78.4 | 0.67 | 0.24 | 0.99 |
| 0.85 | 27.0 | 73.0 | 0.67 | 0.29 | 1.03 |
| 0.88 | 31.5 | 68.5 | 0.67 | 0.33 | 1.06 |
| 0.90 | 35.4 | 64.6 | 0.67 | 0.37 | 1.06 |
| 0.92 | 40.4 | 59.6 | 0.67 | 0.42 | 1.05 |
| 0.95 | 51.0 | 49.0 | 0.67 | 0.54 | 0.92 |

**Table S4.** **Sensitivity of trajectory-segment classification to the inactivity cutoff.** The cutoff was applied to $p_{i}(S)$, defined as the proportion of observed detections within a trajectory segment with speed below the movement threshold. For each cutoff, the table reports the proportion of retained trajectory segments classified as active or inactive, together with median segment duration for each class. Summaries were computed over the analysis period extending from day 6 of life to the day on which female broilers were removed from the house, using retained trajectory segments with at least 30 detections.

| **Cutoff** | **N units** | **Median PI** | **Correlation with PI at 0.90** | **Median absolute difference** | **Mean absolute difference** | **P95 absolute difference** |
| --- | --- | --- | --- | --- | --- | --- |
| 0.80 | 5,663 | 0.838 | 0.982 | 0.135 | 0.128 | 0.184 |
| 0.85 | 5,663 | 0.785 | 0.994 | 0.083 | 0.079 | 0.110 |
| 0.88 | 5,663 | 0.738 | 0.999 | 0.038 | 0.036 | 0.050 |
| 0.90 | 5,663 | 0.698 | 1.000 | 0.000 | 0.000 | 0.000 |
| 0.92 | 5,663 | 0.644 | 0.998 | 0.049 | 0.047 | 0.064 |
| 0.95 | 5,663 | 0.524 | 0.978 | 0.153 | 0.151 | 0.200 |

**Table S5. Sensitivity of observation-unit PI to the inactivity cutoff.** For each alternative cutoff, PI was recalculated at the 90-min observation-unit level and compared with PI obtained using the reference cutoff of 0.90. The table reports the Pearson correlation with the reference PI, the median and 95th percentile of the absolute difference in PI, and the median PI under each cutoff. Summaries were computed using the same final filters as in Table S3.


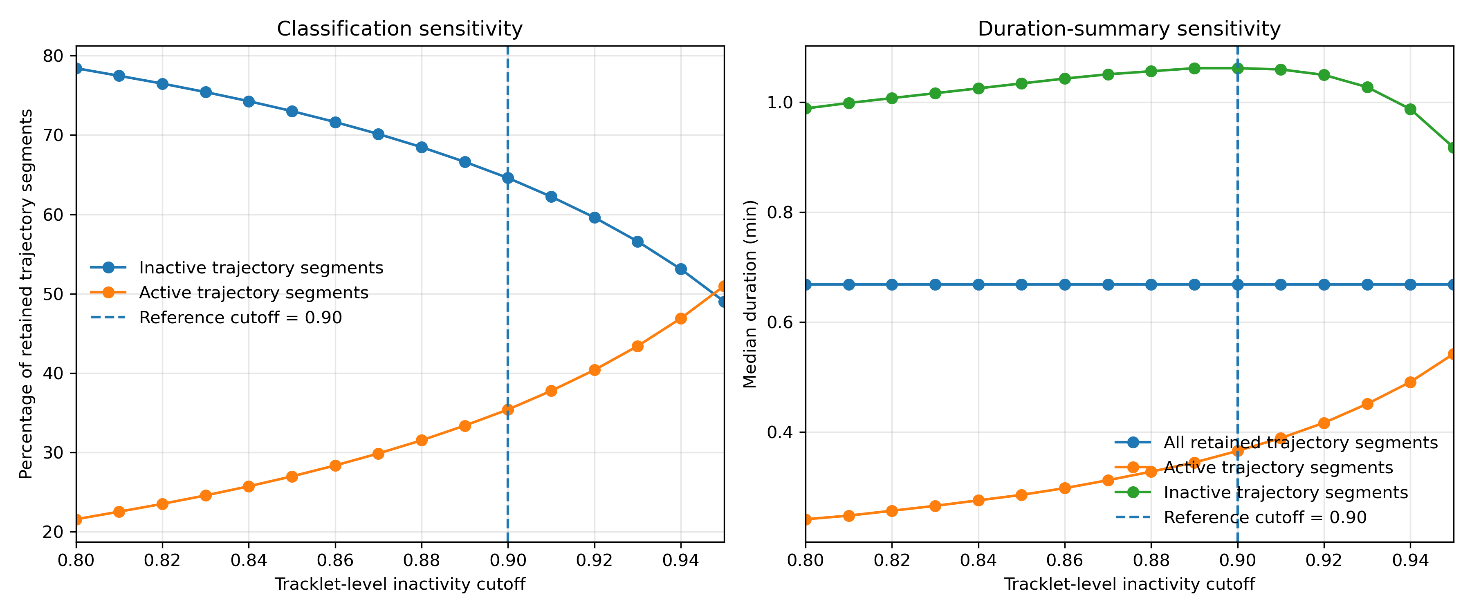


**Figure S3. Sensitivity of trajectory-segment classification to the inactivity cutoff.** The left panel shows the percentage of retained trajectory segments classified as active or inactive across alternative inactivity cutoffs. The right panel shows the corresponding median duration of all retained, active, and inactive trajectory segments. The dashed vertical line indicates the reference cutoff of 0.90 used in the main analysis.


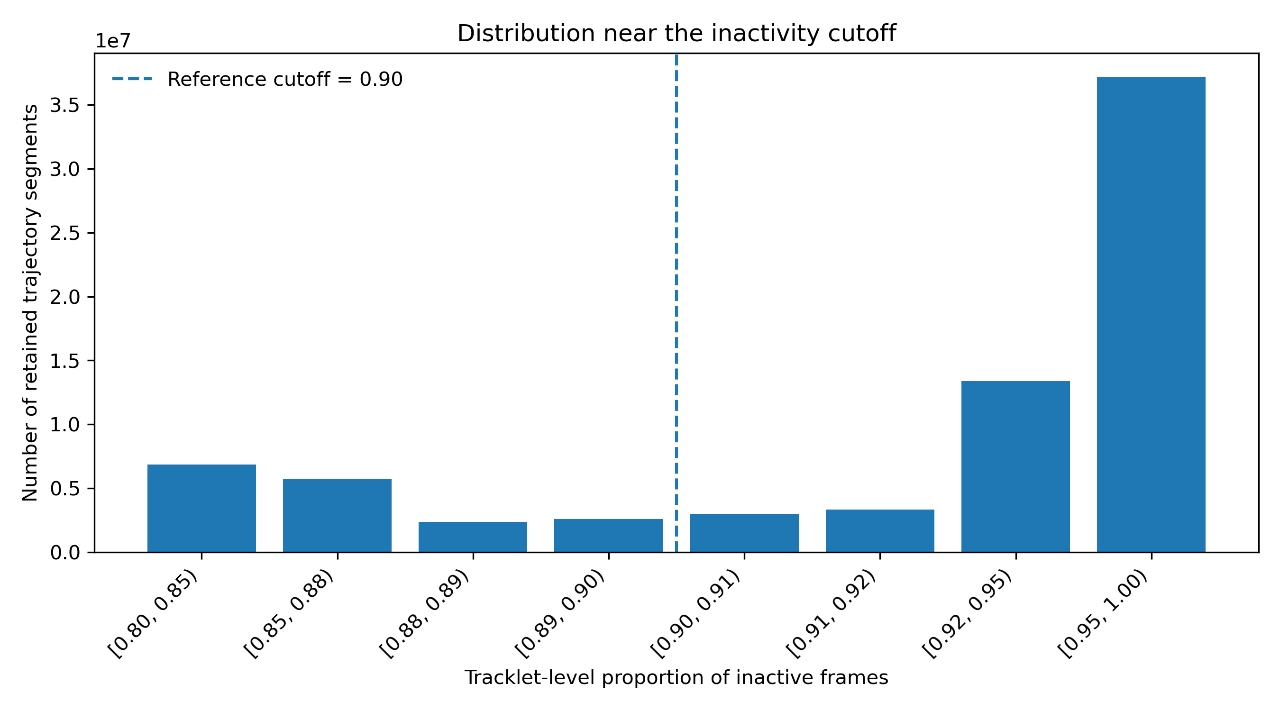


**Figure S4. Distribution of trajectory segments around the reference inactivity cutoff.** Bars show the number of retained trajectory segments according to their proportion of detections below the movement threshold, using intervals around the reference cutoff of 0.90. The dashed vertical line indicates the cutoff used in the main analysis.

**S3.2 Model formulation and interaction assessments**

| **Model** | **Formula** | **df** | **R^2^** | **Adj. R^2^** | **Residual SE** | **AIC** |
| --- | --- | --- | --- | --- | --- | --- |
| Base model | PI_logit ~ day + period + sex + farm | 9 | 0.799 | 0.799 | 0.304 | 1372.6 |

**Table S6.** Base linear model for inactivity (logit-transformed proportion of inactivity, $PI_{w}$). The model includes additive effects of day of life, day period, sex, and farm, and serves as the reference for interaction testing.

| **Interaction tested** | $\boldsymbol{\Delta}\boldsymbol{df}$ | **F** | **p-value** | $\boldsymbol{\Delta}\boldsymbol{AIC}$ |
| --- | --- | --- | --- | --- |
| Day x Farm | 3 | 26.31 | 8.6×10⁻¹⁷ | −72.2 |
| Day x Sex | 1 | 56.05 | 9.3×10⁻¹⁴ | −53.7 |
| Sex x Farm | 3 | 9.27 | 4.2×10⁻⁶ | −21.8 |
| Period x Sex | 2 | 11.69 | 8.8×10⁻⁶ | −19.4 |
| Period x Farm | 6 | 3.96 | 6.0×10⁻⁴ | −11.8 |

**Table S7.** Statistical assessment of interaction terms for the inactivity model. Each interaction was tested against the base model using partial F-tests. ΔAIC values are reported relative to the base model; negative values indicate improved fit.

| **Model** | **Interactions included** | **df** | **R^2^** | **Adj. R^2^** | **Residual SE** | **AIC** |
| --- | --- | --- | --- | --- | --- | --- |
| Full model | day×farm, period×farm, sex×farm, day×sex, period×sex | 24 | 0.813 | 0.811 | 0.294 | 1196.6 |
| Reduced model | day×farm, day×sex | 13 | 0.808 | 0.807 | 0.298 | 1247.8 |

**Table S8.** Summary of model complexity and goodness-of-fit metrics for the full interaction model including all screened and the reduced model retaining only the selected interactions based on parsimony and predictive performance.

| **Comparison** | $\boldsymbol{\Delta}\boldsymbol{df}$ | **F** | **p-value** |
| --- | --- | --- | --- |
| Full vs. Reduced | 11 | 6.69 | 3.5×10⁻¹¹ |

**Table S9.** Formal comparison between the full and reduced interaction models using a partial F-test. Despite a statistically significant difference, the reduced model was retained due to its lower complexity and similar explanatory power.


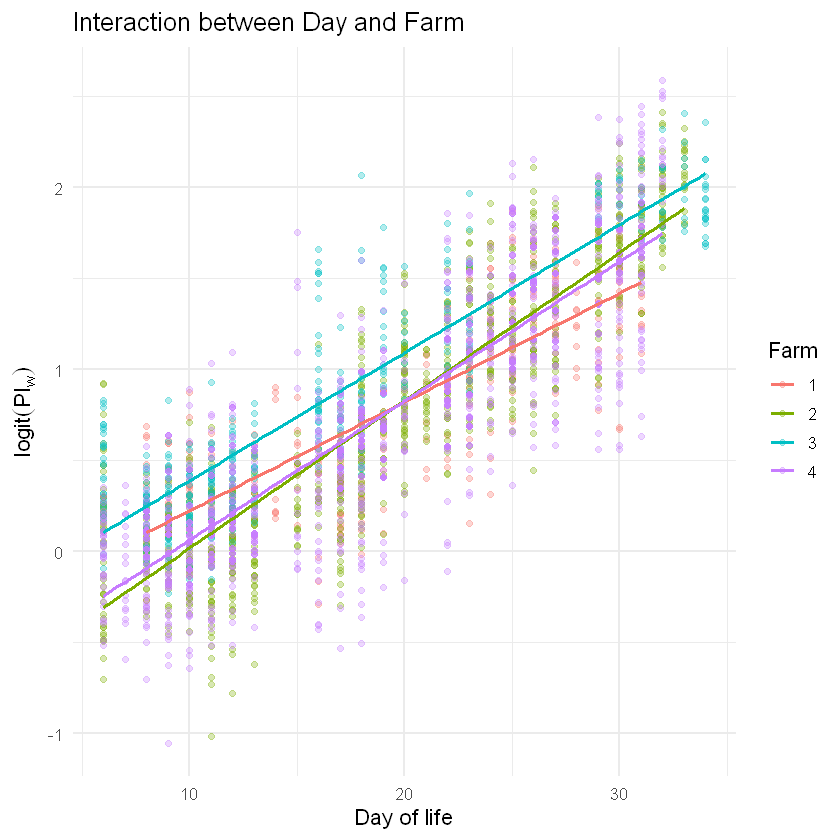


**Figure S5.** Interaction between day of life and farm for inactivity. Points represent individual observations of logit-transformed inactivity ($PI_{w}$). Solid lines correspond to linear fits estimated separately for each farm.


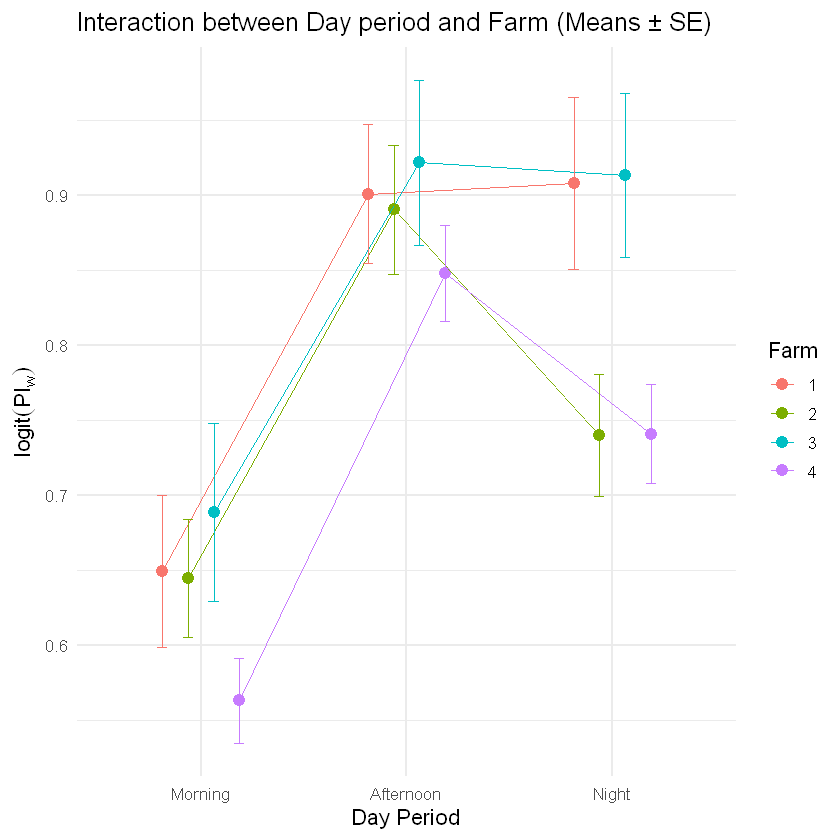


**Figure S6.** Interaction between day period and farm for inactivity. Points and lines represent mean inactivity (± SE) within each farm and day period.


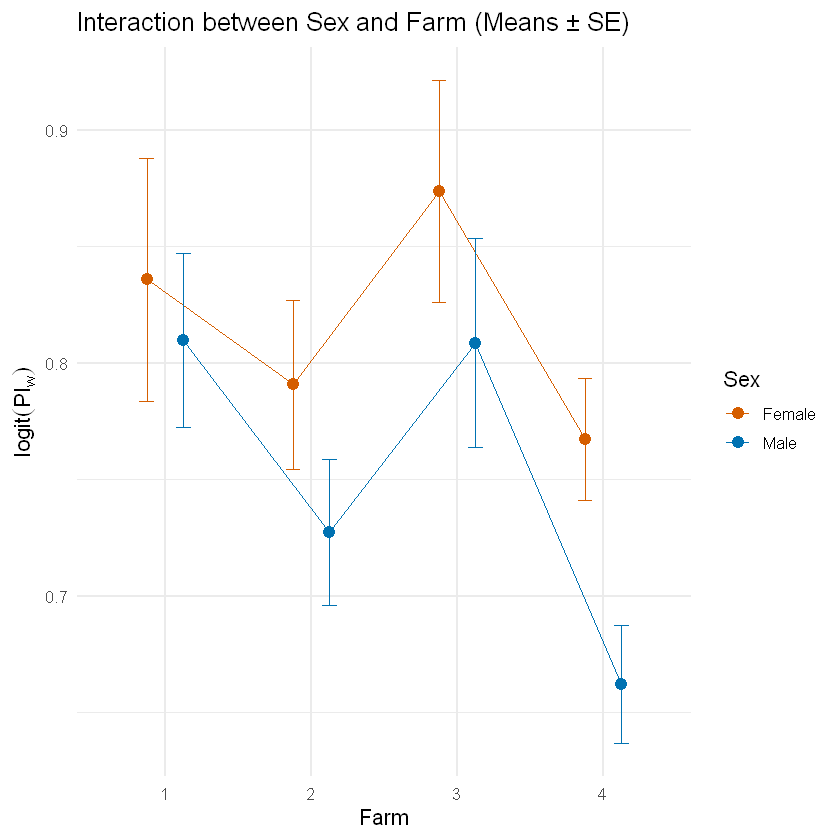


**Figure S7.** Interaction between sex and farm for inactivity. Mean logit-transformed inactivity (± SE) is shown for females and males within each farm.


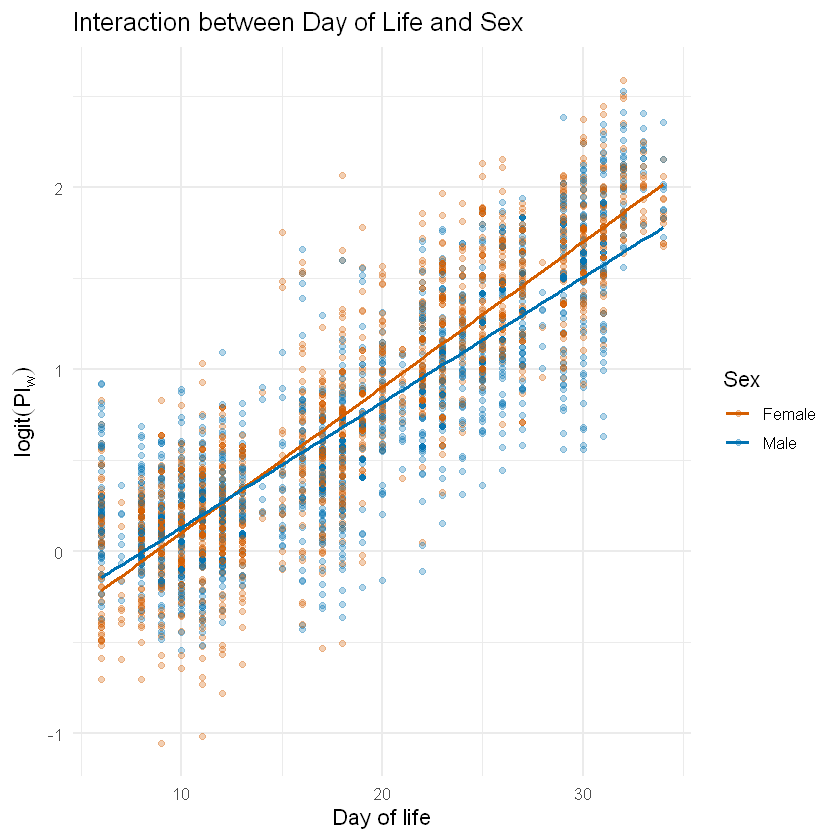


**Figure S8.** Interaction between day of life and sex for inactivity. Points represent individual observations, and lines show sex-specific linear trends over time.


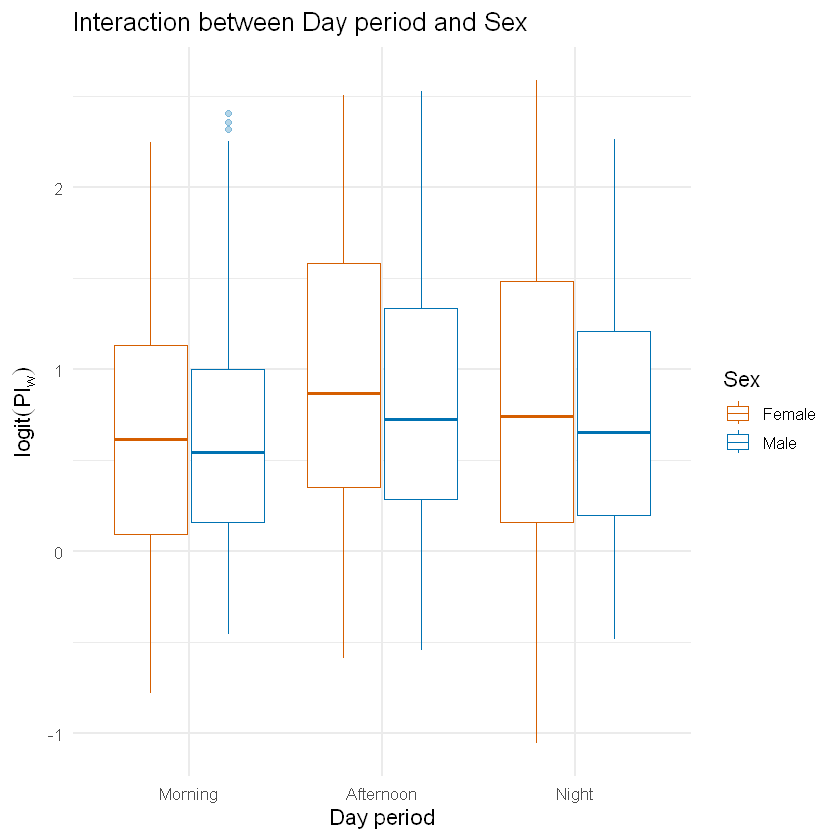


**Figure S9.** Interaction between day period and sex for inactivity. Boxplots summarize the distribution of logit-transformed inactivity for females and males across day periods.

**S3.3 Diagnostics for the reduced model**

Residual diagnostics for the reduced model were conducted to assess the validity of the underlying model assumptions (Figures S10). Visual inspection of the residuals versus fitted values (Figure S10a) revealed no strong systematic pattern, supporting the assumptions of linearity and homoscedasticity. This visual assessment was consistent with the Non-Constant Variance Score Test, which provided no evidence of heteroscedasticity ${(X}^{2}=0.15, df=1, p=0.697)$. The normal Q–Q plot of standardized residuals (Figure S10b) showed that residuals closely follow the theoretical normal distribution in the central region, with mild deviations observed in the tails. Although the Shapiro-Wilk test indicated a statistically significant departure from normality $(W = 0.9948, p < 0.0001)$, this result is expected given the large sample size and does not indicate a practically relevant violation of model assumptions. The histogram of residuals (Figure S10c) further illustrates an approximately symmetric distribution centered around zero. Finally, the Cook’s distance plot (Figure S10d) showed no observations with undue influence on the fitted model, as no values stood out relative to the overall distribution. Taken together, these diagnostic results support the adequacy and robustness of the reduced model for the analyses presented.


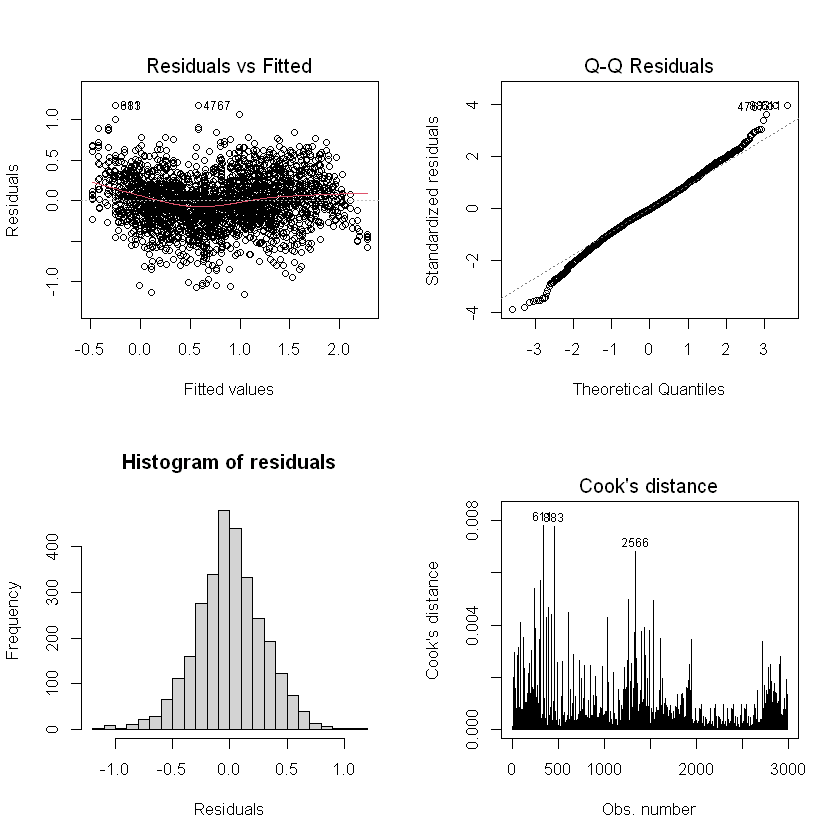


**Figure S10. Diagnostic plots for the final reduced linear model fitted to inactivity (**$logit(PI_{w})$**).** (a) Residuals versus fitted values, showing no strong systematic pattern and indicating approximate homoscedasticity; the red curve corresponds to a LOESS smoother. (b) Normal Q–Q plot of standardized residuals, with residuals closely following the theoretical normal distribution and only minor deviations in the tails. (c) Histogram of residuals, illustrating an approximately symmetric distribution centered around zero. (d) Cook’s distance for all observations, indicating no influential points exceeding conventional thresholds. Overall, these diagnostics support the adequacy of the reduced model assumptions.

**S3.4 Comparison of alternative model strategies**

| **Model** | **Formulation** | **CV scheme** | **RMSE (train)** | **R² (train)** | **RMSE(CV)** | **R² (CV)** |
| --- | --- | --- | --- | --- | --- | --- |
| Reduced | day + farm + sex + period + day:farm + day:sex | Stratified-farm | **0.298** | **0.808** | **0.298 ± 0.010** | **0.806 ± 0.013** |
|  |  | Stratified-flock |  |  | **0.298 ± 0.010** | **0.805 ± 0.016** |
|  |  | Grouped- flock |  |  | 0.323 ± 0.055 | 0.772 ± 0.031 |
| Mixed (1\|farm) | day + sex + period + day:sex + (1\|farm) | Stratified-farm | 0.301 | 0.772† | 0.301 ± 0.010 | 0.802 ± 0.012 |
|  |  | Stratified- flock |  |  | 0.302 ± 0.010 | 0.801 ± 0.016 |
|  |  | Grouped- farm |  |  | 0.341 ± 0.038 | 0.709 ± 0.093 |
|  |  | Grouped- - flock |  |  | **0.314 ± 0.045** | **0.784 ± 0.016** |
| Farm-agnostic | day + sex + period + day:sex | Stratified-farm | 0.315 | 0.784 | 0.316 ± 0.010 | 0.783 ± 0.013 |
|  |  | Stratified- flock |  |  | 0.316 ± 0.009 | 0.782 ± 0.017 |
|  |  | Grouped-farm |  |  | **0.334 ± 0.042** | **0.721 ± 0.090** |
|  |  | Grouped- flock |  |  | 0.319 ± 0.064 | 0.778 ± 0.041 |

**Table S10.** Performance comparison of alternative modelling strategies for group-level inactivity (PI_logit).
RMSE and R² are reported for training fits and under multiple cross-validation (CV) schemes, including stratified and grouped designs based on farm and flock. CV results are shown as mean ± standard deviation across folds. Rows are color-coded according to the cross-validation scheme to facilitate comparison across modelling strategies: same color indicates the same CV design (Stratified–farm, Stratified– flock, Grouped–farm, Grouped– flock). This visual grouping highlights how model performance varies as a function of the validation strategy rather than the model formulation alone.

†For mixed-effects models, training R² corresponds to the marginal R² (R²ₘ), reflecting variance explained by fixed effects only. The mixed model with random slopes (1+day|farm) was excluded due to convergence issues.

**S3.5 Prediction interval and alert detection in the non-routine dataset**

This section reports a quantitative summary of alert detection when the 95% prediction intervals derived from the reduced inactivity model were applied to the non-routine dataset. Alerts correspond to observations whose $PI_{logit}$ values fell outside the expected range defined under routine conditions.

| **Farm** | **Sex** | $\boldsymbol{n}_{\boldsymbol{total}}$ | $\boldsymbol{n}_{\boldsymbol{alert}}$ | **Proportion of alerts** |
| --- | --- | --- | --- | --- |
| 1 | Female | 112 | 11 | 0.098 |
| 1 | Male | 125 | 12 | 0.096 |
| 2 | Female | 231 | 40 | 0.173 |
| 2 | Male | 231 | 33 | 0.143 |
| 3 | Female | 372 | 19 | 0.051 |
| 3 | Male | 344 | 25 | 0.073 |
| 4 | Female | 295 | 18 | 0.061 |
| 4 | Male | 285 | 31 | 0.109 |
| **Overall** | **-** | **1995** | **189** | **0.0947** |

**Table S11. Alert summary for the non-routine dataset based on prediction intervals of the reduced inactivity model.** The table reports, for each farm × sex combination, the total number of excluded observations ($n_{tot\mathrm{al}}$), the number classified as alerts ($n_{alert}$), and the corresponding proportion of alerts. Alerts are defined as observations whose logit-transformed proportion of inactivity ($PI_{logit}$) lies outside the 95% prediction intervals estimated from data collected under routine-condition. The non-routine dataset consists of video segments removed a priori due to environmental disturbances, technical irregularities, or reported non-routine events potentially affecting normal behavioral expression.

| **Model** | **Excluded alerts (%)** |
| --- | --- |
| Reduced | 9.5 |
| Mixed (1\|farm) | 9.0 |
| Farm-agnostic | 10.3 |

**Table S12.** Proportion of observations classified as alerts in the non-routine dataset for alternative inactivity models. Alerts were defined as observations falling outside the 95% prediction intervals derived from each model, applied without refitting. The non-routine dataset was not used for model training and contains observations recorded under conditions incompatible with the definition of routine conditions.

**S4. Active chickens**

**S4.1 Model specification**

| **Model** | **Formulation** | **df** | | **Dispersion** | **AIC** |
| --- | --- | --- | --- | --- | --- |
| Gamma Fix | day + period + sex | 6 | 0.0757 | | −4987.4 |
| Gamma Fix-Farm | day + period + sex + farm | 9 | 0.0713 | | −5164.4 |
| Gamma Smooth | bs(day)*period + sex + farm | 17 | 0.0607 | | −5642.7 |
| Gamma Sex-Smooth | bs(day)*period + sex + bs(day):sex + farm | 20 | 0.0598 | | −5682.6 |
| Gamma Farm-Smooth | bs(day)*period + sex + bs(day):farm + farm | 26 | 0.0568 | | −5823.2 |
| Gamma Both-Smooth | bs(day)*period + sex + farm + bs(day):sex + bs(day):farm | 29 | 0.0561 | | −5859.8 |
| Gamma Mixed (1\|farm) | bs(day)*period + bs(day)*sex + (1\|farm) | 18 | 0.0598 | | −5666.9 |
| Gamma Mixed (bs(day)\|farm) | bs(day)*period + bs(day)*sex + (bs(day)\|farm) | 27 | 0.0563 | | NA |

**Table S13.** Alternative Gamma model formulations evaluated for the window-level mean speed of active trajectory segments ($Y_{w}$). The table reports model complexity (df), the Gamma dispersion parameter (σ²) estimated by the model, and AIC. The random smooth-by-farm model (bs(day)|farm) produced convergence warnings and did not return a valid AIC in the fitted output.

**S4.2 Comparison of alternative model strategies**

| **Model** | **CV Scheme** | **R² (train)** | **RMSE (train)** | **R² (CV)** | **RMSE (CV)** |
| --- | --- | --- | --- | --- | --- |
| Gamma Fix | Grouped_farm | 0.660 | 0.149 | 0.672 ± 0.026 | 0.150 ± 0.028 |
|  | Grouped_flock |  |  | 0.660 ± 0.127 | 0.155 ± 0.037 |
|  | Stratified_farm |  |  | 0.658 ± 0.021 | 0.149 ± 0.005 |
|  | Stratified_ flock |  |  | 0.658 ± 0.024 | 0.149 ± 0.002 |
| Gamma Fix-farm | Grouped_ flock | 0.689 | 0.142 | 0.650 ± 0.142 | 0.152 ± 0.035 |
|  | Stratified_farm |  |  | 0.687 ± 0.023 | 0.143 ± 0.005 |
|  | Stratified_ flock |  |  | 0.687 ± 0.023 | 0.143 ± 0.001 |
| Gamma Smooth | Grouped_ flock | 0.739 | 0.130 | 0.698 ± 0.109 | 0.144 ± 0.037 |
|  | Stratified_farm |  |  | 0.737 ± 0.020 | 0.131 ± 0.006 |
|  | Stratified_flock |  |  | 0.735 ± 0.026 | 0.131 ± 0.003 |
| Gamma Sex-Smooth | Grouped_ flock | 0.743 | 0.129 | 0.700 ± 0.109 | 0.144 ± 0.037 |
|  | Stratified_farm |  |  | 0.740 ± 0.019 | 0.130 ± 0.006 |
|  | Stratified_flock |  |  | 0.738 ± 0.028 | 0.131 ± 0.004 |
| Gamma Farm-Smooth | Grouped_flock | 0.748 | 0.128 | 0.674 ± 0.113 | 0.149 ± 0.042 |
|  | Stratified_farm |  |  | 0.744 ± 0.022 | 0.129 ± 0.006 |
|  | Stratified_flock |  |  | 0.742 ± 0.025 | **0.129 ± 0.003** |
| Gamma Both-Smooth | Grouped_flock | **0.751** | **0.127** | 0.677 ± 0.114 | 0.148 ± 0.043 |
|  | Stratified_farm |  |  | **0.747 ± 0.022** | **0.128 ± 0.007** |
|  | Stratified_flock |  |  | **0.745 ± 0.026** | **0.129 ± 0.003** |
| Gamma Mixed (1\|farm) | Grouped_flock | 0.743 | 0.129 | **0.704 ± 0.042** | **0.138 ± 0.025** |
|  | Stratified_farm |  |  | 0.700 ± 0.109 | 0.144 ± 0.037 |
|  | Stratified_flock |  |  | 0.740 ± 0.019 | 0.130 ± 0.006 |

**Table S14.** Cross-validation performance of alternative Gamma model formulations for the window-level mean speed of active trajectory segments. Model performance is reported as RMSE and R^2^ (mean ± standard deviation) under different cross-validation (CV) schemes. Stratified CV preserves the distribution of the grouping variable (farm or production flock) across folds, whereas grouped CV enforces complete separation between training and validation sets, representing more stringent generalization scenarios. Rows are color-coded according to the cross-validation scheme to facilitate comparison across modelling strategies: same color indicates the same CV design (Stratified–farm, Stratified– flock, Grouped–farm, Grouped– flock).

**S4.3 Empirical validation of the Gamma distribution assumptions**

The adequacy of the Gamma distribution for modelling the mean speed of active birds ($Y_{w}$) was assessed using empirical mean-variance relationships and residual diagnostics. All analyses were conducted on the same response variable and aggregation scheme used for model fitting, without redefining observational units or refitting the model.

Under the adopted parametrization, the Gamma model implies a quadratic relationship between the conditional variance and the conditional mean:

$V\left( Y_{w} | x_{w} \right)=\phi\mu_{w}^{2}$,

where $\mu_{w}=E(Y_{w}|x_{w})$ and $\phi$ is the dispersion parameter.

To evaluate this assumption empirically, observations were grouped into cells defined by farm x sex x period x day. Replications within each cell arose from multiple cameras and/or flocks. For each cell, the empirical mean $E\left( Y_{w} \right)$ and the empirical variance $Var(Y_{w})$ were computed.

Figure S11 shows the relationship between the empirical variance and the squared empirical mean. A no-intercept regression of $Var(Y_{w})$ on $E\left( Y_{w} \right)^{2}$ yielded an estimated dispersion parameter $\hat{\phi}=0.051$, with a coefficient of determination $R^{2}=0.63$. The results indicate a clear positive association between variance and squared mean, consistent with the quadratic mean–variance structure implied by the Gamma distribution, although with substantial dispersion at higher mean values.


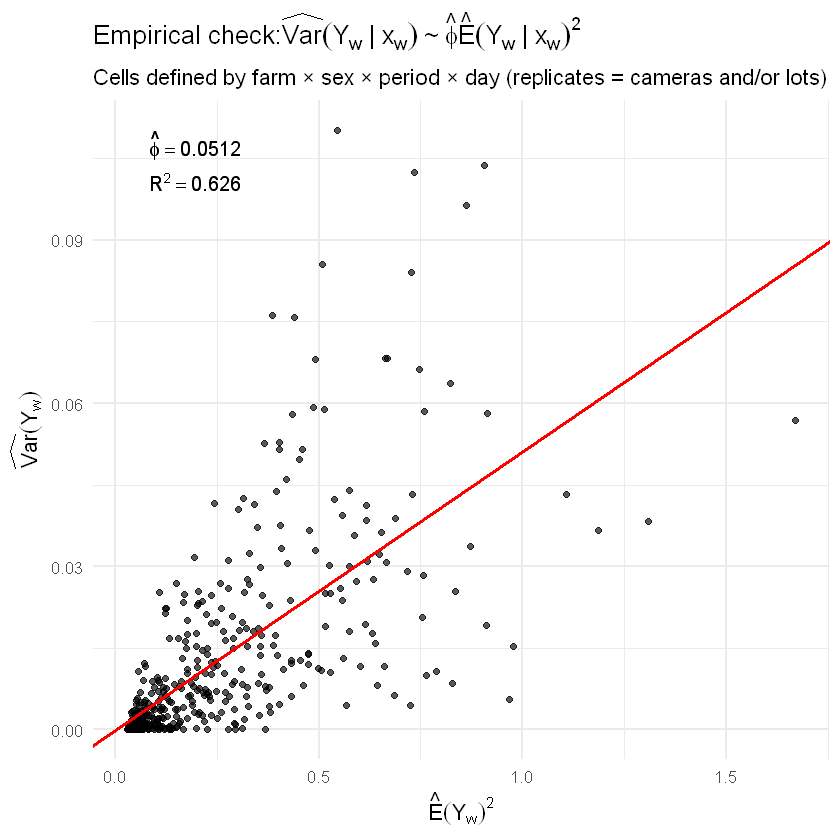


**Figure S11.** Empirical assessment of the Gamma mean–variance relationship for the window-level mean speed of active trajectory segments $(Y_{w})$. Each point corresponds to a cell defined by farm × sex × period × day, with replication arising from multiple cameras and/or flocks. The empirical variance of ${Var(Y}_{w})$ is plotted against the squared empirical mean $E\left( Y_{w} \right)^{2}$. The red line shows the fitted no-intercept regression $Var(Y_{w})=\hat{\phi}E\left( Y_{w} \right)^{2}$, with $\hat{\phi}=0.051$ and $R^{2}=0.63$. The positive association supports a variance structure approximately proportional to the squared mean, as implied by the Gamma distribution.

As a complementary analysis, the mean–variance relationship was examined on a log–log scale (Figure S12). Under an exact Gamma variance function, a slope of 2 would be expected. The estimated slope was steeper $(\hat{b}=3.74)$, indicating that variance increases faster than quadratically with the mean over the observed range. This pattern suggests mild overdispersion relative to the ideal Gamma assumption, particularly at higher activity levels, while preserving a strong monotonic relationship between mean and variance.


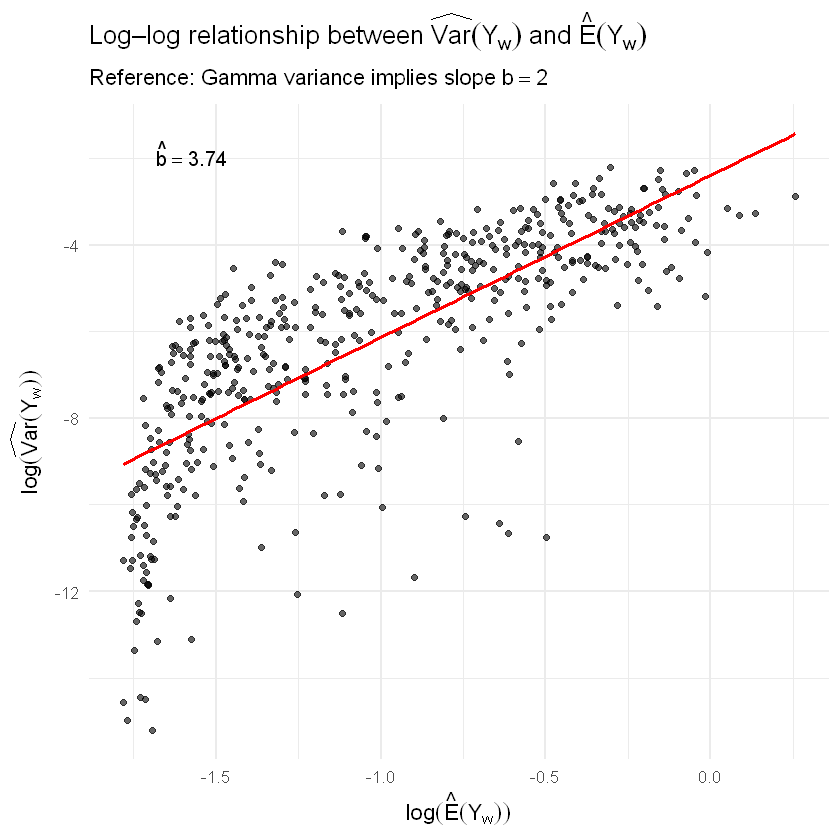


**Figure S12.** Log–log relationship between the empirical variance $Var(Y_{w})$ and the empirical mean $E(Y_{w})$ of the window-level mean speed of active trajectory segments. Points correspond to farm × sex × period × day cells, with replication arising from multiple cameras and/or flocks. The red line shows the fitted linear regression on the log-log scale. Under the canonical Gamma variance function, a slope of 2 is expected. The estimated slope $(\hat{b}=3.74)$ indicates that empirical variance increases faster than quadratically with the mean, consistent with mild overdispersion relative to the ideal Gamma assumption.

Finally, Pearson residuals $r_{w}^{(P)}$ from the fitted Gamma-SexSmooth model were inspected (Figure S13). Residuals were centered around zero across the range of fitted conditional means$\hat{\mu}_{w}$, indicating no major systematic bias in the mean structure. For low fitted means, residuals show a mild positive skew, with more values concentrated near zero and a longer right tail, consistent with the asymmetric support of the Gamma distribution. Residual dispersion became more symmetric for moderate and high values of $\hat{\mu}_{w}$, with no clear systematic trend across the fitted range.


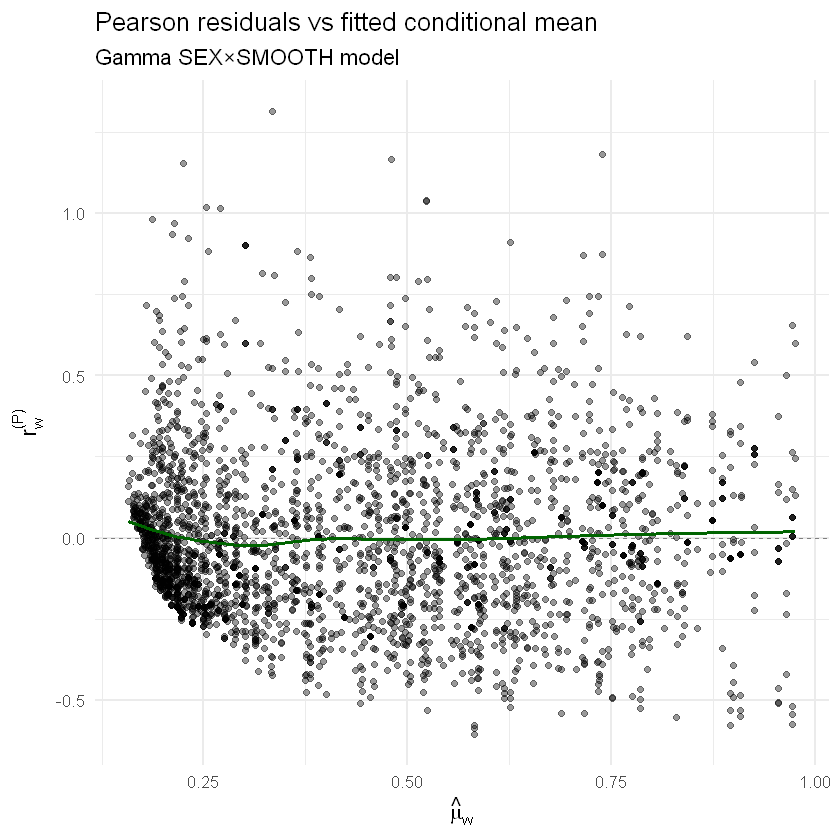


**Figure S13.** Pearson residuals $r_{w}^{(P)}$ ​versus fitted conditional means $\hat{\mu}_{w}$ from the Gamma–SexSmooth model. Each point corresponds to a single observation window $w$. Residuals are centered around zero across the fitted range, with no strong systematic trend. For low fitted means, residuals display a mild positive skew and slightly increased dispersion, consistent with the asymmetric support of the Gamma distribution. Residual variability remains approximately stable for moderate and high values of $\hat{\mu}_{w}$, supporting the adequacy of the model for prediction-interval construction.

Overall, these diagnostics indicate that although the empirical variance of $Y_{w}$ departs from exact quadratic scaling, the Gamma distribution with a log link provides a reasonable and robust approximation of the observed mean–variance structure. Remaining deviations are consistent with biological and observational heterogeneity inherent to commercial production settings and do not compromise the model’s primary objective of defining prediction intervals for deviation-based behavioral surveillance.

**S4.4 Prediction interval and alert detection in the non-routine dataset**

| **Farm** | **Sex** | $\boldsymbol{n}_{\boldsymbol{total}}$ | $\boldsymbol{n}_{\boldsymbol{alert}}$ | **Proportion of alerts** |
| --- | --- | --- | --- | --- |
| 1 | Female | 112 | 4 | 0.036 |
| 1 | Male | 125 | 7 | 0.056 |
| 2 | Female | 231 | 35 | 0.152 |
| 2 | Male | 231 | 40 | 0.173 |
| 3 | Female | 372 | 30 | 0.081 |
| 3 | Male | 344 | 27 | 0.079 |
| 4 | Female | 295 | 22 | 0.075 |
| 4 | Male | 285 | 19 | 0.067 |
| **Overall** | **-** | **1995** | **184** | **0.0922** |

**Table S15.** Number and proportion of observations classified as alerts in the non-routine dataset based on the 95% prediction intervals of the Gamma–SexSmooth model. Alerts correspond to mean speed values falling outside the expected range defined from the routine-condition dataset. Results are summarized by farm and sex. The non-routine dataset includes video segments recorded under conditions incompatible with the definition of routine conditions (e.g., environmental disturbances, technical irregularities, or suspected but not confirmed health impairments); alert classification indicates deviations from the modelled behavioral baseline and does not imply confirmed welfare problems.

**S5. Detection model performance**

| **Model** | **P** | **R** | **mAP50** | **Flops (G)** |
| --- | --- | --- | --- | --- |
| YOLOv8n | 0.95 | 0.92 | 0.96 | 8.1 |
| YOLOv9t | 0.95 | 0.92 | 0.96 | 7.6 |
| YOLOv10n | 0.99 | 0.52 | 0.76 | 8.2 |
| YOLOv11n | 0.95 | 0.91 | 0.96 | 6.3 |
| YOLOv11s | 0.95 | 0.93 | 0.96 | 21.3 |
| YOLOv11m | 0.96 | 0.92 | 0.97 | 67.6 |
| YOLOv12n | 0.95 | 0.91 | 0.96 | 6.3 |

**Table S16.** Performance of different YOLO models used for broiler detection.

- **Precision (P)** is the proportion of predicted detections that correspond to true positives (i.e., correct detections of chickens):

$$\text{Precision}= \frac{\text{True Positives}}{\text{True Positives}+\text{False positives}}$$

- **Recall (R)** is the proportion of actual chickens that were successfully detected:

$$\text{Recall}= \frac{\text{True Positives}}{\text{True Positives}+\text{False negatives}}$$

- **Intersection-over-Union (IoU)** is used to match predictions with ground-truth annotations. For a predicted bounding box $B_{p}$ and a ground-truth box $B_{gt}$, it is defined as:

$\text{IoU} = \frac{\text{Area}\left( B_{p} \cap B_{\mathrm{gt}} \right)}{\text{Area}\left( B_{p} \cup B_{\mathrm{gt}} \right)}$

- **mAP50** corresponds to the mean average precision when $\text{IoU} \geq0.5$. It is computed as the area under the precision–recall curve evaluated at different confidence thresholds, using a fixed IoU threshold of 0.5.
- **FLOPs (Floating Point Operations)** represent the computational cost per image. Models with fewer FLOPs are more efficient for real-time or large-scale deployment.
